# Timing of aneurysm repair and clinical outcomes after aneurysmal subarachnoid hemorrhage: a systematic review

**DOI:** 10.1007/s00415-026-14063-x

**Published:** 2026-09-09

**Authors:** Táya Figueiredo de Oliveira Fiore, Ângela Maria Arantes Vieira, Fábio de Freitas Magalhães, Carolina Guimarães de Souza, Cesar Augusto Ferreira Alves Filho, Thiago Barboza

**Affiliations:** 1 Faculdade de Medicina, Universidade do Grande Rio Professor José de Souza Herdy (Afya – UNIGRANRIO), Duque de Caxias, RJ 25075-142 Brazil; 2 Endovascular Neurosurgery Service, Instituto Estadual Do Cérebro Paulo Niemeyer (IECPN), Rio de Janeiro, RJ CEP 20231-092 Brazil; 3 Vascular Neurosurgery Service, Hospital Municipal Souza Aguiar (HMSA), Rio de Janeiro, RJ CEP 20211-350 Brazil

**Keywords:** Onset-to-treatment, Time-to-treatment, Clipping, Endovascular coiling, Ruptured intracranial aneurysm, ASAH, Complications

## Abstract

**Background:**

Aneurysmal subarachnoid hemorrhage (aSAH) causes substantial morbidity and mortality, and guidelines recommend aneurysm repair as early as feasible. However, the association between onset-to-treatment time and outcomes remains uncertain. This review synthesized evidence across multiple clinical outcomes and examined whether treatment modality modifies this association.

**Methods:**

Searches were conducted in PubMed/MEDLINE, Scopus, and LILACS for studies comparing clinical outcomes across different onset-to-treatment windows in adults with confirmed aSAH. Findings were synthesized narratively according to the Synthesis Without Meta-analysis (SWiM) guideline. The review was prospectively registered in PROSPERO (CRD420261415084).

**Results:**

Twenty reports comprising 11,096 participant records were included, with likely overlap between two reports. Treatment categories ranged from <6 h to ≥15 days. Earlier securement likely reduced pretreatment rebleeding, particularly when untreated or markedly delayed patients were included, although treated-cohort comparisons were inconsistent. More methodologically informative adjusted analyses showed no reproducible independent association between treatment timing and functional outcome or mortality. No consistent association emerged for vasospasm or related cerebral ischemia, hydrocephalus, or length of stay. Two observational studies modeled time continuously: one found a significant U-shaped mortality association with an estimated nadir at 32.6 h, whereas the other showed a similar but non-significant adjusted pattern with an estimated nadir near 12.16 h. These findings are highly susceptible to confounding by indication and survivor bias and do not establish benefit from treatment delay. Three studies formally tested modality-timing interaction; one found a significant mortality interaction and two did not. Additional stratified analyses showed no consistent modality-specific pattern. Certainty of evidence for the timing-mortality association was very low because of serious risk of bias, inconsistency, and imprecision.

**Conclusion:**

Earlier aneurysm securement remains supported for preventing pretreatment rebleeding. The independent association of treatment timing with mortality or functional outcome remains uncertain. The observed mortality patterns are hypothesis-generating and do not define a validated therapeutic window, support intentional treatment delay, or justify changing current guideline recommendations. Prospective multicenter studies using continuous-time modeling and rigorous methods to address confounding and survivor bias are needed.

**Supplementary Information:**

The online version contains supplementary material available at https://doi.org/10.1007/s00415-026-14063-x.

## Introduction

Aneurysmal subarachnoid hemorrhage (aSAH) is a severe form of stroke caused by rupture of an intracranial aneurysm, carrying high morbidity and mortality despite representing only 5% of all cerebrovascular events and up to 27% of stroke-related years of potential life lost [1]. Global incidence varies substantially by region, and the socioeconomic burden is compounded by aSAH disproportionately affecting individuals in their fifth and sixth decades, with significant long-term functional disability among survivors [2]. Clinical severity is commonly assessed using the Hunt-Hess and WFNS scales, while the extent of hemorrhage is quantified by the Fisher scale; these scores guide therapeutic decisions and predict complications such as vasospasm, delayed cerebral ischemia (DCI), and rebleeding [3].

Definitive treatment aims to exclude the aneurysm through microsurgical clipping or endovascular embolization. Current international guidelines recommend intervention as early as feasible, ideally within 24 h of onset, to improve outcomes [2]. However, the relationship between onset-to-treatment interval and clinical outcomes appears more complex than a simple linear benefit of earlier intervention. Recent meta-analyses demonstrate that ultra-early treatment (≤ 6 h) may reduce rebleeding risk but are underpowered to determine effects on survival or functional recovery, while early treatment (≤ 12 h) has paradoxically been associated with increased rebleeding risk [4].

Furthermore, confounding by indication, whereby patients with higher clinical severity (high WFNS or Hunt-Hess grades) are treated more urgently, complicates the interpretation of observational data, and the apparent benefit of early treatment often attenuates after adjustment for baseline severity. The heterogeneity of time cutoffs used across studies (variably defined as within 24, 48, or 72 h) and the variable inclusion of high-grade patients have further prevented a clear consensus [2].

Therefore, this systematic review aims to synthesize the available evidence on the association between onset-to-treatment time and immediate outcomes (mortality, functional outcome, complications) in aSAH, while also examining whether the treatment modality (clipping vs. coiling) modifies this relationship. By identifying consistent patterns and methodological pitfalls, we seek to provide a practical framework for future research and for optimizing neurovascular care networks across different healthcare settings.

## Materials and methods

### Study design and registration

This systematic review was prospectively registered in PROSPERO (CRD420261415084) and followed the PRISMA 2020 [5] and Synthesis Without Meta-analysis (SWiM) guidelines [6]. The review question was structured according to PICOS: Population – adults with confirmed aneurysmal subarachnoid hemorrhage (aSAH); Intervention/Exposure – onset-to-treatment time for microsurgical clipping or endovascular coiling; Comparison – different time windows (< 24 h, 24–72 h, >72 h, and finer cutoffs when reported); Outcomes – mortality, functional outcome (mRS or GOS), pretreatment rebleeding, vasospasm/related cerebral ischemia, hydrocephalus, and length of stay; Study design – prospective or retrospective cohort studies, case–control studies, and randomized controlled trials. Full-text articles in English, Portuguese, or Spanish were included; studies without a clear temporal definition, narrative or systematic reviews, case reports, and non-indexed publications were excluded. The certainty of evidence for mortality, the primary outcome, was assessed using GRADE (Supplementary Table S1). As all included studies were observational regarding treatment timing, certainty started at low and was downgraded to very low, the lowest GRADE category, for serious risk of bias. Serious inconsistency and imprecision reinforced this judgment without further downgrading; no upgrading factors were identified.

### Search strategy

Initial searches were conducted between January and March 2026 in PubMed/MEDLINE, Scopus, and LILACS and updated in LILACS on 15 April 2026, Scopus on 17 April 2026, and PubMed/MEDLINE on 18 April 2026; no additional eligible studies were identified. The search strategy targeted studies on aneurysm repair timing. Filters excluded reviews and non-human trials and limited publication to 2015–2026. The complete database-specific search strategies are provided in Supplementary Table S2.

### Study selection criteria

**Fig. 1:**
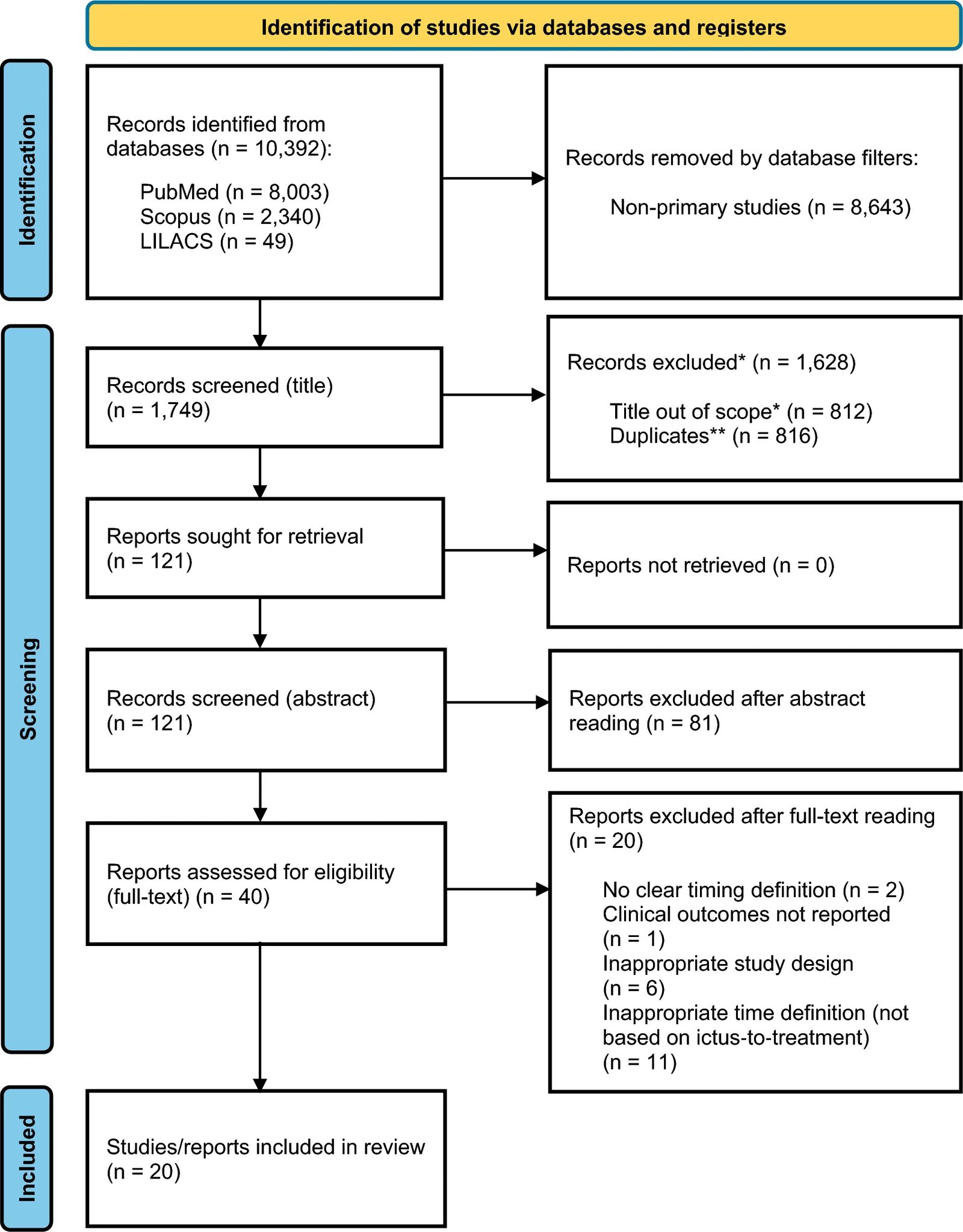
PRISMA flow diagram of the study selection process. *Title-level screening was assisted by a generative AI tool (large language model) applying PICO-based exclusion criteria. Two reviewers manually verified all AI-flagged records before exclusion. Full criteria are described in the Study selection criteria section. **Duplicates across PubMed, Scopus, and LILACS were removed using Mendeley and manually verified

During title-level screening, a generative AI tool (Anara AI, large language model, March 2026) was used exclusively as a pre-filter. The exclusion criteria in Supplementary Table S3 were structured as a PICO-based prompt applied to each title and available metadata: “If ANY criterion is clearly met, classify as EXCLUDE. If insufficient information to decide, classify as RETAIN – erroneously retaining a borderline record is preferable to erroneously excluding a potentially eligible one.” Of the 1749 records screened at title level, 812 were flagged by the AI as meeting at least one exclusion criterion and excluded only after manual confirmation by two reviewers. After these exclusions and removal of 816 duplicates, the remaining 121 records underwent independent abstract screening by two human reviewers using the predefined eligibility criteria. The same two reviewers then independently assessed the retrieved full-text reports to confirm that they addressed onset-to-treatment time and the clinical outcomes of interest. Disagreements at the abstract and full-text stages were resolved by consensus or consultation with a third reviewer. Ultimately, 20 studies met the inclusion criteria and were included (Fig. 1). Formal inter-rater agreement statistics were not calculated because item-level independent screening decisions before consensus were not retained in a format permitting valid retrospective estimation.

### Data extraction and quality assessment

Two reviewers independently extracted data using a standardized form, resolving discrepancies by consensus or a third reviewer. Extracted data included study characteristics (author, year, country, design, sample size); demographics (age, sex); clinical severity (WFNS, Hunt-Hess, Fisher grades); intervention parameters (onset-to-treatment interval, clipping vs. coiling); and outcomes (functional status by modified Rankin Scale or Glasgow Outcome Scale, mortality, rebleeding, vasospasm/related cerebral ischemia, hydrocephalus, and length of stay).

Two reviewers independently assessed methodological limitations, resolving disagreements by consensus. Because onset-to-treatment time was not randomized in any study, including the two post hoc analyses of randomized trial data, all were evaluated as observational timing studies. An item-level, domain-based framework derived from the Newcastle–Ottawa Scale assessed cohort representativeness, comparison-group selection, treatment-timing ascertainment, control of baseline clinical severity and other confounders, outcome assessment, and follow-up completeness. Timing-specific concerns – confounding by indication, survivor bias, and selective inclusion of treated survivors – were recorded separately. Domain judgments were presented without score summation or global low-, moderate-, or high-risk labels.

### Data analysis

Given the expected heterogeneity of timing thresholds, findings were synthesized using a broad temporal framework (< 24 h, 24–72 h, and >72 h) for qualitative comparison. Study-specific timing categories were preserved; results were not reassigned when reported categories did not align with these boundaries, and finer or alternative definitions were retained in the narrative synthesis and tables. Only outcome data linked to treatment timing through categorical groups or continuous-time models contributed to timing comparisons; global reporting without a temporal comparison was recorded separately. Baseline variables presented across treatment-timing groups were retained as descriptive timing-based data and explicitly identified as baseline characteristics. Results were additionally stratified by modality (clipping vs. coiling) to examine whether the time-outcome association differs by treatment type.

Alternative quantitative approaches were considered infeasible given the available data. Dose–response meta-analysis was precluded by incompatible, frequently open-ended timing categories, insufficient event reporting across exposure levels, and heterogeneous effect measures. Meta-regression was considered unreliable because few studies contributed usable timing-based data to each outcome, amid substantial study-level heterogeneity in baseline severity, treatment modality, and analytical adjustment. Harmonized continuous-time modeling was not possible without individual participant data; only two studies modeled onset-to-treatment time continuously, whereas the remaining studies reported aggregate data using study-specific categorical thresholds. Findings were therefore synthesized narratively following the SWiM guideline [6].

In the narrative synthesis, analyses adjusting for baseline clinical severity and other major confounders received greater interpretive weight. Propensity-adjusted, propensity-matched, and comprehensively adjusted multivariable analyses were considered more methodologically informative than crude, descriptive, subgroup-only, or unadjusted comparisons, although residual confounding and survivor bias could remain after adjustment. Descriptive findings were considered hypothesis-generating, particularly without formal comparisons or with small event counts, and did not determine the synthesis direction.

Effect measures were retained as reported and explicitly identified as odds ratios, risk ratios, hazard ratios, or regression coefficients. Because these measures estimate different quantities and were based on different outcome codings, follow-up periods, and reference categories, their magnitudes were not compared directly across studies. The modeled outcome and reference category were reported wherever available.

### Ethical aspects

Data was not collected directly from patients or medical records. All information was accessed from studies previously approved by research ethics committees.

## Results

Twenty reports published in 2015–2026 included 11,096 participant records with confirmed aSAH (Table 1). The reports by Wang B. et al. [7, 8] used the same multicenter database and recruitment period and likely included substantially overlapping participants; therefore, the total does not represent 11,096 unique individuals. The studies comprised 18 retrospective cohorts and two post hoc analyses of data from multicenter randomized controlled trials [9, 10]. Because treatment timing was not randomized in the post hoc analyses, both were observational regarding the exposure of interest. Twelve studies were single-center and eight multicenter. Ten originated from Asia, six from Europe, two from South America, one from North America, and one from Oceania.

**Table 1:** Characteristics of included studies

Author (year); Title	Country/Continent	Design & Setting	*N*	Population	Mean age/Sex (% female)	Treatment modality	Onset-to-treatment groups
*Brawanski, 2020 *[11]	Germany/Europe	Retrospective cohort, single center	449	Poor-grade aSAH (WFNS IV–V)	55.4 ± 13.5/64.1%	Microsurgery 179 (39.9%), endovascular 225 (50.1%), no aneurysm repair 45 (10.0%)	0–12 h/13–24 h/25–35 h/≥36 h; <12 h vs. >12 h (descriptive)
*Buscot, 2022 *[12]	Australia/Oceania	Retrospective cohort, multicenter	482	First-ever aSAH (2010–2016)	55.0 ± 14.5/69.9%	Microsurgery 186 (38.6%), Endovascular 296 (61.4%)	Continuous (hours); sensitivity analysis ≤72 h
*Dellaretti, 2018 *[13]	Brazil/South America	Retrospective cohort, single center	218	aSAH (2008–2016)	50.0 ± 11.2/71.1%	Microsurgery only: 218 (100%)	0–2 d/3–10 d/>10 d
*Hostettler, 2023 *[14]	Germany/Europe	Retrospective cohort, single center	853	aSAH (2006–2020)	57.3 ± 14.3/66.6%	Microsurgery 314 (36.8%), Endovascular 539 (63.2%)	<24 h/>24 h
*Koester, 2023 *[15]	USA/North America	Retrospective cohort, single center	1013	aSAH (2014–2019)	56 ± 14/69%	Microsurgery 595 (58.7%), Endovascular 418 (41.3%)	≤24 h/>24 h
*Kurisu, 2023 *[16]	Japan/Asia	Retrospective cohort, single center	215	aSAH (2015–2021)	64.5 ± 15.0/67.0%	Both: Microsurgery 133 (61.9%), Endovascular 82 (38.1%)	≤24 h/>24 h; subacute: days 1–3/4–10/day 15
*Lage, 2020 *[17]	Portugal/Europe	Retrospective cohort, single center	343	aSAH (2000–2016)	56 (IQR 45–68)/64.7%	Microsurgery only: 343 (100%)	<24 h/24–72 h
*Loconi-Vallejos, 2023 *[18]	Peru/South America	Retrospective cohort, single center	132	aSAH (2018–2021)	52.5 ± 15.0/69.7%	Microsurgery only: 132 (100%)	1–3 d/4–10 d/>10 d
*Lu, 2023 *[19]	China/Asia	Retrospective cohort, multicenter	127	Poor-grade aSAH (HH IV–V; 2020–2022)	60 (IQR 51–65)/57.5%	Microsurgery 60 (47.2%), Endovascular 67 (52.8%)	0–2 d/3–10 d/>10 d
*Ohbuchi, 2023 *[20]	Japan/Asia	Retrospective cohort, multicenter	253	Poor-grade aSAH (WFNS IV–V; 2010–2020)	63.6 ± 14.6/64%	Microsurgery 102 (40.3%), Endovascular 126 (49.8%), no aneurysm repair 25 (9.9%)	≤13 h/>13–72 h/>72 h (appropriate treatment); <24 h/24–72 h/>72 h/no repair
*Şimsek, 2022 *[21]	Turkey/Europe-Asia	Retrospective cohort, single center	212	Poor-grade aSAH (WFNS IV–V; 2001–2020)	58.3 ± 13.7/55.2%	Microsurgery only: 96 (45.3%), no aneurysm repair 116 (54.7%)	<6 h/6–72 h/>72 h/no repair
*Teo, 2017 *[10]	UK/Europe	Post hoc analysis of RCT, multicenter	767	aSAH, age 18–65, STASH trial (2007–2013)	50 (range 20–69)/68.4%	Microsurgery 254 (33.1%), Endovascular 513 (66.9%)	<24 h/25–48 h/49–96 h/>96 h
*Tykocki, 2017 *[22]	Poland/Europe	Retrospective cohort, single center	79	Poor-grade aSAH (WFNS IV–V; 2011–2013)	61.8 ± 7.5/57.0%	Microsurgery 47 (59.5%), Endovascular 32 (40.5%)	<24 h/>24 h
*Vergouwen, 2023 *[9]	Netherlands/Europe	Post hoc analysis of RCT, multicenter	497	aSAH treated within 24 h of onset (2013–2019)	57 (IQR 49–65)/71%	Microsurgery 109 (21.9%), Endovascular 388 (78.1%)	<6 h/6–24 h
*Wang B., 2025 *[7]	China/Asia	Retrospective cohort, multicenter	990	Poor-grade aSAH (WFNS IV–V; 2017–2020)	60.8 ± 11.2/66.5%	Microsurgery 441 (44.5%), Endovascular 549 (55.5%)	<12 h/12–23 h/≥24 h
*Wang B., 2026 *[8]	China/Asia	Retrospective cohort, multicenter	3560	aSAH treated within 72 h of onset (2017–2020)	58.7 ± 10.8/65.3%	Microsurgery 1258 (35.3%), Endovascular 2302 (64.7%)	<12 h/12-<25 h/25–<42 h/≥42 h
*Wu, 2022 *[23]	China/Asia	Retrospective cohort, single center	86	Poor-grade aSAH (HH IV–V; 2018–2021)	58.9 ± 8.9 (< 24 h); 61.3 ± 8.2 (> 24 h)/60.5%	Endovascular only: 86 (100%)	<24 h/>24 h
*Zhang F., 2017 *[24]	China/Asia	Retrospective cohort, single center	280	aSAH (2012–2014)	51.7 ± 10.8/57.9%	Endovascular only: 280 (100%)	Within 12 h/≥12 h
*Zhang X., 2021*[25]	China/Asia	Retrospective cohort, single center	422	aSAH (2017–2019)	56.8 ± 12.0/65.2%	Endovascular only: 422 (100%)	1 st day/2–3 d/4–14 d/≥15d
*Zhao, 2015 *[26]	China/Asia	Retrospective cohort, multicenter	118	Poor-grade aSAH (WFNS IV–V; 2010–2014)	54.9 ± 11.5/57.6%	Microsurgery only: 118 (100%)	<24 h/>24 h

*HH* Hunt–Hess grade, *WFNS* World Federation of Neurosurgical Societies grade, *RCT* randomized controlled trial. In Brawanski et al. [11], reliable ictus time was available for 211 patients. The inconsistently reported third interval was retained as the figure-based category of 25–35 h. Teo et al. [10] and Vergouwen et al. [9] were post hoc RCT analyses with nonrandomized treatment timing. Reports by Wang B. et al. [7, 8] used the Chinese multicenter Cerebral Aneurysm Database, with the same 12 centers and 2017–2020 recruitment period; participant overlap was likely substantial but unquantifiable from published reports

Reported mean or median age ranged from 50.0 to 64.5 years, with most estimates between 55 and 60 years. Female sex predominated in all studies (55.2–71.1%). Baseline clinical severity was assessed using the WFNS, Hunt-Hess, and/or Fisher scales. Twelve studies included both microsurgical clipping and endovascular treatment, five included clipping only, and three included endovascular treatment only. Onset-to-treatment categories ranged from <6 h to ≥15 days; four studies primarily categorized timing in days rather than hours (Table 1).

Several study-specific timing categories crossed the broad temporal framework boundaries and could not be reassigned to <24 h, 24–72 h, or >72 h without altering their original meaning. These studies were therefore retained and interpreted using their original timing definitions in the outcome-specific tables.

Table 2 summarizes treatment-timing associations by outcome. Of the 20 studies, the numbers reporting each outcome and contributing timing-based analyses, respectively, were: functional outcome, 19/19; mortality, 15/12; rebleeding, 16/12; vasospasm/related cerebral ischemia, 13/9; hydrocephalus, 12/7; and length of stay, 5/3.

**Table 2:** Outcome reporting and availability of timing-based analyses

Outcome (studies reporting)	Reporting category/*n*	Studies
*Functional outcome (mRS/GOS) (19)*	Only reported 0	
	Timing-based analyses 19	Brawanski, 2020 [11]; Dellaretti, 2018 [13]; Hostettler, 2023 [14]; Koester, 2023 [15]; Kurisu, 2023 [16]; Lage, 2020 [17]; Loconi-Vallejos, 2023 [18]; Lu, 2023 [19]; Ohbuchi, 2023 [20]; Şimsek, 2022 [21]; Teo, 2017 [10]; Tykocki, 2017 [22]; Vergouwen, 2023 [9]; Wang B., 2025 [7]; Wang B., 2026 [8]; Wu, 2022 [23]; Zhang F., 2017 [24]; Zhang X., 2021 [25]; Zhao, 2015 [26]
*Mortality (15)*	Only reported 3	Ohbuchi, 2023 [20]; Teo, 2017 [10]; Zhang X., 2021 [25]
	Timing-based analyses 12	Buscot, 2022 [12]; Dellaretti, 2018 [13]; Hostettler, 2023 [14]; Koester, 2023 [15]; Lage, 2020 [17]; Şimsek, 2022 [21]; Tykocki, 2017 [22]; Vergouwen, 2023 [9]; Wang B., 2025 [7]; Wang B., 2026 [8]; Wu, 2022 [23]; Zhao, 2015 [26]
*Rebleeding (16)*	Only reported 4	Loconi-Vallejos, 2023 [18]; Lu, 2023 [19]; Teo, 2017 [10]; Wu, 2022 [23]
	Timing-based analyses 12	Brawanski, 2020 [11]; Buscot, 2022 [12]; Hostettler, 2023 [14]; Koester, 2023 [15]; Lage, 2020 [17]; Ohbuchi, 2023 [20]; Tykocki, 2017 [22]; Şimsek, 2022 [21]; Vergouwen, 2023 [9]; Wang B., 2025 [7]; Wang B., 2026 [8]; Zhao, 2015 [26]
*Vasospasm/related cerebral ischemia (13)*	Only reported 4	Brawanski, 2020 [11]; Loconi-Vallejos, 2023 [18]; Ohbuchi, 2023 [20]; Teo, 2017 [10]
	Timing-based analyses 9	Buscot, 2022 [12]; Hostettler, 2023 [14]; Koester, 2023 [15]; Kurisu, 2023 [16]; Lu, 2023 [19]; Şimsek, 2022 [21]; Vergouwen, 2023 [9]; Wang B., 2026 [8]; Zhao, 2015 [26]
*Hydrocephalus (12)*	Only reported 5	Brawanski, 2020 [11]; Loconi-Vallejos, 2023 [18]; Lu, 2023 [19]; Ohbuchi, 2023 [20]; Teo, 2017 [10]
	Timing-based analyses 7	Buscot, 2022 [12]; Hostettler, 2023 [14]; Kurisu, 2023 [16]; Şimsek, 2022 [21]; Wang B., 2026 [8]; Wu, 2022 [23]; Zhao, 2015 [26]
*Length of stay (5)*	Only reported 2	Brawanski, 2020 [11]; Teo, 2017 [10]
	Timing-based analyses 3	Koester, 2023 [15]; Tykocki, 2017 [22]; Wang B., 2025 [7]

Values indicate the number of studies reporting the outcome/number contributing a timing-based analysis. Global outcome reporting and baseline characteristics without a temporal comparison were classified as “only reported.” Timing-based analyses included categorical comparisons, continuous-time models, and qualitative timing–outcome findings without numerical estimates

Across all 20 reports, selection into timing analyses was of high concern, most commonly because analyses were restricted to treated survivors, excluding untreated patients, pretreatment deaths, or those without complete timing data. Control of additional confounders was frequently incomplete, particularly for referral and diagnostic delays, clinical instability, treatment logistics, and physician-driven treatment selection (Supplementary Table S4). Accordingly, conclusions relied primarily on propensity-adjusted or multivariable analyses with stronger control of baseline severity and clinically relevant confounders. Crude, descriptive, or subgroup-only comparisons and adjusted models with major specification or post-exposure-adjustment concerns were retained for completeness but given less interpretive weight. Descriptive findings, particularly without formal comparisons or with small event counts, were treated as hypothesis-generating. Assessment times varied; outcomes measured at discharge and different follow-up periods were not considered equivalent or directly comparable.

### Primary outcomes

*Functional outcome.* Nineteen studies contributed timing-based findings assessed from discharge to a median follow-up of 34.3 months. Because functional status may change during recovery, Table 3 groups studies as favoring earlier treatment, later treatment, no difference, or non-significant numerical trends; these directional categories summarize study-specific findings and do not imply equivalence across assessment times.

**Table 3:** Association between onset-to-treatment time and functional outcome after aSAH

Study	Population	Outcome, assessment time & treatment window	Early % (n/N)	Late % (n/N)	Effect measure (95% CI)	Analysis type
*Favoring earlier treatment*						
* Brawanski, 2020 *[11]	Poor-grade	mRS 0–2 (6 mo) [favorable], ≤12 h vs. >12 h	45.8% (38/83)	35.9% (46/128)	No formal statistical comparison or effect estimate reported	Descriptive
* Lage, 2020 *[17]	All grades	GOS 4–5 (6 mo) [favorable], <24 h vs. 24–72 h	82.4% (136/165)	71.9% (128/178)	Reported HR 3.21 (95% CI 1.56–6.61; *p* = 0.002), reference not stated	Multivariable-adjusted
* Lu, 2023 *[19]	Poor-grade	mRS 4–6 (3 mo) [unfavorable], 0–2 d (ref.) vs. 3–10 d	46.4% (39/84)	72.2% (26/36)	aOR 3.06 (95% CI 1.27–7.39); *p* = 0.013	Multivariable-adjusted
* Lu, 2023 *[19]	Poor-grade	mRS 4–6 (3 mo) [unfavorable], 0–2 d (ref.) vs. >10 d	46.4% (39/84)	85.7% (6/7)	aOR 6.59 (95% CI 0.66–66.05); *p* = 0.109	Multivariable-adjusted
* Ohbuchi, 2023 *[20]	Poor-grade	mRS 0–3 (3 mo) [favorable], appropriate treatment ≤13 h vs. 13–72 h (ref.);	37% (37/100)	17% (17/98)	aOR 2.74 (95% CI 1.30–5.74); *p* = 0.0064	Multivariable-adjusted
* Simsek, 2022 *[21]	Poor-grade	GOS 4–5 (6 mo) [favorable], <6 h vs. ≥6 h/no repair	50% (17/34)	15.2% (27/178)	No effect estimate reported; *p* < 0.001	Crude/ unadjusted
* Tykocki, 2017 *[22]	Poor-grade	mRS 0–3 (discharge) [favorable], <24 h vs. >24 h	58% (22/38)	22% (9/41)	Crude OR 4.89 (95% CI 1.83–13.03) calculated from the reported event counts	Crude
* Wu, 2022 *[23]	Poor-grade	mRS 0–2 (6 mo) [favorable], <24 h vs. >24 h (ref.)	61.3% (38/62)	25.0% (6/24)	aOR 7.00 (95% CI 1.80–27.24); *p* = 0.005	Multivariable-adjusted
* Zhang F., 2017 *[24]	All grades	GOS 4–5 (3 mo) [favorable], within 12 h vs. ≥12 h (as reported) (ref.)	–	–	aOR 0.38 (95% CI 0.19–0.75); *p* = 0.005	Multivariable-adjusted
*Favoring later treatment*						
* Vergouwen, 2023 *[9]	All grades	mRS 3–6 (6 mo) [unfavorable], <6 h vs. 6–24 h (ref.)	57.3% (63/110)	37.5% (145/387)	aRR 1.36 (95% CI 1.11–1.66)	Multivariable-adjusted
* Wang B., 2025 *[7]	Poor-grade	mRS 3–6 at follow-up (median 34.3 mo) [unfavorable], <12 h (ref.) vs. ≥24 h	–	–	Lower adjusted odds of unfavorable outcome; estimate not reported	Multivariable-adjusted
* Wang B., 2026 *[8]	All grades	mRS 3–5 (2y) [unfavorable], continuous	–	–	Adjusted RCS: trend *p* = 0.040; overall *p* = 0.019; nonlinearity *p* = 0.055; plateau at ~32.6 h; no categorical reference	Multivariable-adjusted RCS
* Zhang X., 2021 *[25]	All grades	mRS 0–2 (discharge) [favorable], onset-to-treatment interval	–	–	Shorter treatment time associated with poorer discharge outcome; counts, effect estimate, *p* value, and reference not reported	Descriptive
*No difference*						
* Hostettler, 2023 *[14]	All grades	GOS 4–5 (discharge) [favorable], <24 h (ref.) vs. >24 h	54.4% (380/698)	74.2% (115/155)	aOR 1.16 (95% CI 0.68–2.04); *p* = 0.580	Multivariable-adjusted
* Koester, 2023 *[15]	All grades	mRS >2 (≥ 180 d) [unfavorable], ≤24 h vs. >24 h (ref.)	22.2% (8/36)	23.9% (85/355)	aOR 0.86 (95% CI 0.31–2.15); *p* = 0.760	Propensity-adjusted
* Teo, 2017 *[10]	All grades	mRS 3–6 (6 mo) [unfavorable], <24 h/25–48 h/49–96 h/>96 h	42.0% (34/81)	27.1% (89/328); 23.9% (68/284); 35.7% (20/56)	Unadjusted omnibus *p* = 0.008; adjusted association reported as non-significant	Crude omnibus; adjusted estimate unavailable
* Zhao, 2015 *[26]	Poor-grade	mRS 0–3 at follow-up (mean 12.5 mo) [favorable], <24 h vs. >24 h (ref.)	34.0% (16/47)	59.2% (42/71)	aOR 0.50 (95% CI 0.200–1.30); *p* = 0.110	Multivariable-adjusted
*Non-significant numerical trends*						
* Dellaretti, 2018 *[13]	All grades	mRS ≤2 (discharge) [favorable], 0–2 d (ref.) vs. >10 d	60.0% (36/60)	70.8% (85/120)	Crude OR 1.62 (95% CI 0.85–3.01) for favorable outcome; *p* = 0.150	Crude/unadjusted
* Kurisu, 2023 *[16]	All grades	mRS 0–2 (3 mo) [favorable], <24 h vs. >24 h (ref.)	57.1% (105/184)	74.2% (23/31)	aOR 0.53 (95% CI 0.12–1.93) for favorable outcome; *p* = 0.355	Multivariable-adjusted
* Loconi-Vallejos, 2023 *[18]	All grades	mRS 4–6 (discharge) [unfavorable], 1–3 d (ref.) vs. 4–10 d	18.2% (2/11)	23.7% (14/59)	aRR 2.95 (95% CI 0.80–10.87) for unfavorable outcome; *p* = 0.105	Multivariable-adjusted
* Loconi-Vallejos, 2023 *[18]	All grades	mRS 4–6 (discharge) [unfavorable], 1–3 d (ref.) vs. >10 d	18.2% (2/11)	22.6% (14/62)	aRR 2.65 (95% CI 0.72–9.79) for unfavorable outcome; *p* = 0.145	Multivariable-adjusted

Dashes indicate unreported timing-specific proportions. Early and Late percentages correspond to the mRS or GOS category and assessment time in each row; bracketed labels identify favorable or unfavorable outcomes. Assessment times in the “Outcome measure, assessment time & treatment window” column ranged from discharge to a median 34.3-month follow-up. Because functional status may change during recovery, directional categories are study-specific and do not imply equivalence or direct comparability across assessment times. Analysis type describes the displayed result, not all source analyses. “Descriptive” indicates no formal comparative effect estimate; “crude/unadjusted,” no confounder adjustment. ORs, RRs, and HRs were retained as reported but estimate different quantities and are not numerically comparable. Reference categories are given when reported; omnibus and continuous-time analyses may lack a single categorical reference. Brawanski et al. [11]: the >12-h group pooled the three later categories. Ohbuchi et al. [20]: the 13-h cutoff was data-derived using the Youden index; “appropriate treatment” comprised aneurysm repair and any required intracranial-pressure-control procedure. Şimsek and Akinci [21]: the comparator combined patients treated at ≥6 h and those receiving no aneurysm repair. Teo et al. [10]: the unadjusted four-group comparison was omnibus, without a single reference; the authors reported loss of significance after multivariable adjustment, but no adjusted timing estimate, confidence interval, p value, or reference. Tykocki et al. [22]: adjusted timing estimates were internally inconsistent (text: OR 4.14, 95% CI 3.82–4.35; regression table: OR 2.14, 95% CI 1.82–2.35); the crude OR and 95% CI were therefore calculated from published event counts. Lage et al. [17]: the source labeled the logistic-regression estimate as an HR; this terminology was retained. Zhang F. et al. [24]: “within 12 h” and “≥12 h” overlap at 12 h. The published regression coefficient was inconsistent with the reported OR, but the OR (0.38; 95% CI 0.19–0.75), Wald statistic, and Table 4 p value (0.005) were mutually consistent and retained; the Results text reported p = 0.038. Zhang X. et al. [25]: shorter onset-to-treatment time was associated with poorer discharge outcome, but no timing-specific counts, effect estimate, or p value was reported. *aOR* adjusted odds ratio, *aRR* adjusted risk ratio, *GOS* Glasgow Outcome Scale, *HR* hazard ratio, *mRS* modified Rankin Scale, *OR* odds ratio, *RCS* restricted cubic spline, *RR* risk ratio

Eight studies favored earlier treatment, largely in poor-grade cohorts (WFNS IV-V or Hunt-Hess IV-V). Among adjusted analyses, Wu et al. [23] (aOR 7.00 for <24 h vs >24 h) and Lu et al. [19] (aOR 3.06 for days 3–10 vs days 0–2) reported significant associations favoring earlier treatment; adjusted associations were also reported by Ohbuchi et al. [20], Lage et al. [17], and Zhang F. et al. [24]. Brawanski et al. [11] descriptively favored treatment ≤12 h over >12 h without a formal comparison; this finding was treated as hypothesis-generating. In Ohbuchi et al., however, the association involved the composite “appropriate treatment” within 13 h, whereas aneurysm repair within 24 h versus 24–72 h alone was not significant. Tykocki et al. [22] reported an internally inconsistent adjusted estimate; Table 3 therefore presents the crude OR of 4.89 calculated from published counts. The large crude difference in Şimsek and Akinci [21] used a comparator combining later-treated and untreated patients and was unadjusted.

Four studies favored later treatment, mostly in all-grade cohorts in which greater baseline severity among earlier-treated patients may have influenced the findings. Vergouwen et al. [9] reported worse outcome with treatment <6 h versus 6–24 h (aRR 1.36), although the association attenuated after reclassification or exclusion of patients with rebleeding. Wang B. et al. [7] reported lower adjusted odds of unfavorable outcome with treatment ≥24 h than <12 h. In Wang B. et al. [8], the risk of 2-year dependent survival decreased with increasing treatment time, most steeply within the first 12 h, and plateaued at approximately 32.6 h; this differed from the U-shaped mortality association. Zhang X. et al. [25] reported a qualitative association between shorter treatment time and poorer outcome at discharge but provided no timing-specific estimate, p value, or counts; only onset-to-admission time remained independently associated in the multivariable model.

Four studies found no independent association after adjustment. Crude associations in Hostettler et al. [14], Teo et al. [10], and Zhao et al. [26] disappeared or attenuated after adjustment, while Koester et al. [15] reported a null propensity-adjusted analysis. Loconi-Vallejos et al. [18] showed a non-significant adjusted trend favoring earlier treatment, whereas Kurisu et al. [16] and Dellaretti et al. [13] showed non-significant trends favoring later treatment.

Overall, the more methodologically informative analyses were predominantly null and did not demonstrate a reproducible independent benefit of earlier treatment. Significant adjusted associations favoring earlier treatment arose in studies with concerns related to treated-survivor selection, incomplete confounder control, composite or data-derived exposure definitions, post-exposure adjustment, or internally inconsistent reporting. Significant findings in either direction remained vulnerable to residual confounding or incomplete follow-up; crude and descriptive associations therefore did not determine the conclusion.

*Mortality.* Fifteen studies reported mortality; 12 provided timing-based analyses (Table 4). Mortality was assessed in hospital or at discharge, at 6 or 12 months, or later; Lage et al. [17] did not report the assessment time. Because mortality is cumulative, findings across periods were not treated as equivalent. Of the 12 analyses, two favored earlier treatment, three favored later treatment, four found no difference, and three showed non-significant trends. Proportions ranged from 3.3% at discharge in Zhang X. et al. [25] to 78.7% at 6 months in the later-treated/untreated comparator of Şimsek and Akinci [21], but were not directly comparable because populations and assessment periods differed.

**Table 4:** Association between onset-to-treatment time and mortality

Study	Population	Outcome, assessment time & treatment window	Early % (n/N)	Late % (n/N)	Effect measure (95% CI)	Analysis type
*Favoring earlier treatment*						
* Şimsek, 2022 *[21]	Poor-grade	Mortality at 6 mo, <6 h vs. ≥6 h/no repair (ref.)	38.2% (13/34)	78.7% (140/178)	Crude OR 0.17; *p* < 0.001	Crude/unadjusted
* Tykocki, 2017 *[22]	Poor-grade	Mortality at discharge, <24 h vs. >24 h	13.2% (5/38)	34.1% (14/41)	No effect estimate; *p* = 0.023; reference not reported	Crude/unadjusted
*Favoring later treatment*						
* Vergouwen, 2023 *[9]	All grades	Mortality at 6 mo, <6 h vs. 6–24 h (ref.)	26.4% (29/110)	14.0% (54/387)	aRR 1.57 (95% CI 1.07–2.33)	Multivariable-adjusted
* Wang B., 2025 *[7]	Poor-grade	Mortality at follow-up (median 34.3 mo), <12 h (ref.)/12–23 h/≥24 h	46.6% (149/320)	38.2% (84/220); 32.4% (146/450)	12–23 h: aHR 0.73 (95% CI 0.55–0.95), *p* = 0.021; ≥24 h: aHR 0.64 (95% CI 0.51–0.81), *p* < 0.001	Multivariable-adjusted
* Wang B., 2026 *[8]	All grades	Mortality at follow-up (mean 30.9 ± 22.5 mo), continuous	–	–	Adjusted RCS: overall *p* = 0.035; nonlinearity *p* = 0.015; nadir at ~32.6 h; no categorical reference	Multivariable-adjusted
*No difference*						
* Hostettler, 2023 *[14]	All grades	In-hospital mortality, <24 h (ref.) vs >24 h	14.8% (103/698)	9.7% (15/155)	aOR 1.56 (95% CI 0.80–3.03); *p* = 0.190	Multivariable-adjusted
* Koester, 2023 *[15]	All grades	In-hospital mortality or at follow-up (< 180 d), ≤24 h vs >24 h	10.6% (10/94)	13.8% (127/919)	No effect estimate; *p* = 0.390; reference not reported	Crude/unadjusted
* Wu, 2022 *[23]	Poor-grade	Mortality (6 mo) <24 h vs >24 h	8.1% (5/62)	8.3% (2/24)	No formal comparison or effect estimate; reference not reported	Descriptive
* Zhao, 2015 *[26]	Poor-grade	Mortality at follow-up (mean 12.5 mo), <24 h vs >24 h (ref.)	31.9% (15/47)	31.0% (22/71)	aOR 0.70 (95% CI 0.30–1.80); *p* = 0.435	Multivariable-adjusted
*Non-significant numerical trends*						
* Buscot, 2022 *[12]	All grades	Mortality (12 mo), continuous	–	–	Adjusted RCS: nonlinearity *p* = 0.120; nadir at 12.16 h; no categorical reference; trend favoring earlier treatment	Multivariable-adjusted RCS
* Dellaretti, 2018 *[13]	All grades	In-hospital mortality after surgery, 0–2 d (ref.) vs >10 d	20.0% (12/60)	10.0% (12/120)	Crude OR 0.44 (95% CI 0.19–1.06); *p* = 0.070; trend favoring later treatment	Crude/unadjusted
* Lage, 2020 *[17]	All grades	Overall mortality (assessment time not specified), <24 h vs 24–72 h	7.3% (12/165)	13.5% (24/178)	7.3% vs 13.5%; *p* = 0.061; trend favoring earlier treatment	Crude/unadjusted

Dashes indicate unreported timing-specific mortality proportions. Mortality assessment periods, when available, are given in the “Outcome measure, assessment time & treatment window” column and included in-hospital, discharge, 6-month, 12-month, and long-term follow-up; non-reporting is stated explicitly. Because mortality is cumulative, directional categories are study-specific and do not imply equivalence or direct comparability across assessment periods. Analysis type describes the displayed result, not all source analyses. “Descriptive” indicates no formal comparative effect estimate; “crude/unadjusted,” no confounder adjustment. Reference categories are marked “(ref.)” when reported; continuous-time analyses lack a single categorical reference. ORs, RRs, and HRs were retained as reported but estimate different quantities and are not numerically comparable. Koester et al. [15]: mortality included deaths during hospitalization or follow-up; mean (SD) follow-up was 708 (1200) days in the ≤24-h group and 737 (1201) days in the >24-h group. Lage et al. [17]: mortality assessment time was unspecified. Şimsek and Akinci [21]: the comparator combined 62 patients clipped at ≥6 h and 116 receiving no aneurysm repair; the logistic model included no additional covariates. Wu et al. [23]: mortality rates were descriptively similar, without a formal comparison. *aHR* adjusted hazard ratio, *aOR* adjusted odds ratio, *aRR* adjusted risk ratio, *HR* hazard ratio, *OR* odds ratio, *RCS* restricted cubic spline, *RR* risk ratio, *SD* standard deviation

Both crude analyses favoring earlier treatment involved poor-grade cohorts. Tykocki et al. [22] reported lower discharge mortality with treatment <24 h than >24 h, and Şimsek and Akinci [21] lower 6-month mortality with clipping <6 h than in a comparator combining later-treated and untreated patients. Lage et al. [17] showed a non-significant trend favoring earlier treatment. Buscot et al. [12] found a significant unadjusted nonlinear association; the adjusted association was non-significant, although a similar pattern and estimated nadir at approximately 12.2 h remained.

All three analyses favoring later treatment were adjusted. Vergouwen et al. [9] reported higher 6-month mortality with treatment <6 h than 6–24 h (aRR 1.57). Wang B. et al. [7] reported lower mortality hazards at 12–23 h and ≥24 h than <12 h; Wang B. et al. [8] identified a significant U-shaped association with a nadir at approximately 32.6 h. Dellaretti et al. [13] showed a non-significant crude trend favoring treatment >10 days over 0–2 days.

Among studies using a 24-h threshold, Hostettler et al. [14] found no adjusted overall association, although treatment >24 h was associated with mortality in the HH grade 3 subgroup (aOR 3.13). Koester et al. [15] reported no crude difference, and Zhao et al. [26] no adjusted association. Wu et al. [23] descriptively reported similar 6-month mortality without a formal comparison; with only seven deaths, this finding was considered hypothesis-generating.

Three studies reported mortality without timing stratification: Ohbuchi et al. [20] compared treatment completeness, Teo et al. [10] reported deaths by modality, and Zhang X. et al. [25] reported overall discharge mortality.

The evidence does not support a consistent independent association between earlier treatment and reduced mortality. Continuous-time analyses suggested possible nonlinearity, but only Wang B. et al. [8] reported a statistically significant adjusted U-shaped association.

*Rebleeding.* Sixteen studies reported rebleeding; 12 provided timing-based analyses. Pretreatment rebleeding occurs while the aneurysm remains unsecured and is the component most directly linked to delayed securement; postoperative rebleeding instead reflects treatment durability and occlusion quality and was analyzed separately. Mixed or unspecified events were summarized separately and were not considered directly comparable to pretreatment rebleeding.

Two studies including untreated patients reported lower crude rebleeding with earlier treatment. Ohbuchi et al. [20] reported rebleeding before or during repair in 7%, 10%, and 38% of patients treated within 24 h, at 24–72 h, and after 72 h, respectively, and 16% of untreated patients. Rates did not differ between treatment within 24 h and at 24–72 h but were lower with treatment within 72 h than with later or no treatment. The comparison of appropriate treatment completed ≤13 h versus >13–72 h showed a non-significant numerical difference (5% vs 11%). Şimsek and Akinci [21] reported rebleeding in 0%, 17.1%, 22.2%, and 49.1% of patients clipped at <6 h, 6–72 h, >72 h, or not repaired, respectively (omnibus *p* < 0.001), but did not specify whether events occurred before or after repair.

Among treated cohorts, no consistent association emerged. Brawanski et al. [11] reported decreasing crude proportions across the 0–12 h, 13–24 h, 25–35 h, and ≥36 h groups (8%, 4%, 1.5%, and 1%) among 211 treated patients with reliable ictus timing, without a formal comparison or separation of pre- and postoperative events. In the absence of an adjusted analysis, this pattern does not demonstrate a protective effect of delayed treatment and may reflect survivor bias or selective treatment allocation. Buscot et al. [12] and Hostettler et al. [14] reported null adjusted associations; Hostettler specifically assessed pretreatment rebleeding. Koester et al. [15] and Lage et al. [17] found no differences, whereas Tykocki et al. [22] reported numerically fewer radiological signs of rebleeding with treatment <24 h, without a formal comparison. These three studies did not specify event timing relative to repair; Koester also excluded patients who reruptured or died before transfer. Descriptive patterns based on few events were considered hypothesis-generating.

Vergouwen et al. [9] reported higher crude “any rebleeding” with treatment <6 h than 6–24 h (26.4% vs 12.7%) but did not separate pre- and postoperative events. Wang B. et al. [7] reported a low overall incidence and no difference across three treatment windows, without group-specific rates. Wang B. et al. [8] reported a small inverse association between onset-to-treatment time and postoperative rebleeding (aOR 0.985 per hour), susceptible to selection and survivor bias. Zhao et al. [26] found no difference in either pretreatment or postoperative rebleeding. Post-treatment event rates were generally low, and the association reported by Wang B. et al. [8] was not reproduced.

Four studies lacked usable timing-based comparisons: Teo et al. [10] reported rebleeding by treatment modality without separating pre- and postoperative events; Lu et al. [19] qualitatively described more pretreatment events after day 10 without timing-window counts; Loconi-Vallejos et al. [18] reported only an overall rate; and Wu et al. [23] described two pretreatment reruptures at 14 and 55 h without attributing them to timing groups.

Overall, earlier securement likely reduces the opportunity for pretreatment rebleeding, particularly when untreated or markedly delayed patients are included. However, treated-cohort comparisons were inconsistent and commonly limited by selection bias and failure to distinguish pre- from postoperative events.

### Secondary outcomes

*Vasospasm and related cerebral ischemia.* Thirteen studies reported vasospasm, DCI or postoperative cerebral ischemia; nine provided usable timing-based analyses (Table 5). Seven used categorical groups [9, 14–16, 19, 21, 26], whereas Buscot et al. [12] and Wang B. et al. [8] modelled time continuously; four reported only global rates [10, 11, 18, 20].

**Table 5:** Association between onset-to-treatment time and vasospasm/related cerebral ischemia

Study	Population	Outcome definition	Time window	Early % (n/N)	Late % (n/N)	Effect measure (95% CI)	Analysis type
*Favoring earlier treatment*							
* Şimsek, 2022 *[21]	Poor-grade	CVS	<6 h/6–72 h/>72 h/no repair	5.9% (2/34)	25.7% (9/35); 11.1% (3/27); 6.9% (8/116)	Omnibus *p* = 0.018; no reference reported	Crude omnibus
* Lu, 2023 *[19]	Poor-grade	DCI	0–2 d (ref.) vs 3–10 d	32.1% (27/84)	52.8% (19/36)	aOR 2.44 (95% CI 1.06–5.64); *p* = 0.036	Multivariable-adjusted
* Hostettler, 2023 *[14]	Lower-grade (HH 1–3)	Cerebral infarction	<24 h (ref.) vs >24 h	–	–	aOR 1.85 (95% CI 1.01–3.33); *p* = 0.050; HH 3: aOR 7.69 (95% CI 2.44–25.00); *p* < 0.001	Multivariable-adjusted subgroup analysis
*Favoring later treatment*							
* Vergouwen (2023) *[9]	All grades	DCI	<6 h vs 6–24 h	32.7% (36/110)	26.1% (101/387)	No formal comparison or effect estimate	Descriptive
*No difference*							
* Buscot, 2022 *[12]	All grades	DCI	Continuous onset-to-treatment time	–	–	Adjusted OR 1.00 per hour (95% CI 0.98–1.00); nonlinearity *p* = 0.880	Multivariable-adjusted RCS
* Hostettler, 2023 *[14]	All grades	CVS	<24 h vs >24 h	58.2% (406/698)	53.6% (83/155)	No effect estimate; *p* = 0.290; no reference reported	Crude/unadjusted
* Hostettler, 2023 *[14]	All grades	Cerebral infarction	<24 h (ref.) vs >24 h	19.9% (139/698)	15.5% (24/155)	aOR 1.56 (95% CI 0.92–2.70); *p* = 0.100	Multivariable-adjusted
* Koester, 2023 *[15]	All grades	CVS	≤24 h vs >24 h	68.1% (64/94)	70.8% (651/919)	No effect estimate; *p* = 0.660; no reference reported	Crude/unadjusted
* Koester, 2023 *[15]	All grades	DCI-related infarction	≤24 h vs >24 h (ref.)	36.2% (34/94)	36.2% (333/919)	PA-adjusted OR 0.87 (95% CI 0.53–1.42); *p* = 0.600	Propensity-adjusted
* Kurisu, 2023 *[16]	All grades	DIND [unfavorable]	<24 h vs >24 h	7.4% (12/163)	9.7% (3/31)	No formal comparison or effect estimate	Descriptive
* Kurisu, 2023 *[16]	All grades	Delayed angiographic vasospasm	<24 h (ref.) vs >24 h	58.9% (96/163)	51.6% (16/31)	Univariable OR 0.73 (95% CI 0.34–1.60); *p* = 0.430	Univariable
* Wang B., 2026 *[8]	All grades	Postoperative cerebral ischemia	Continuous onset-to-treatment time	–	–	Adjusted OR 1.001 per hour (95% CI 0.995–1.007); *p* = 0.753	Multivariable-adjusted continuous-time model
* Zhao, 2015 *[26]	Poor-grade	Cerebral infarction	<24 h vs >24 h	19.1% (9/47)	18.3% (13/71)	No effect estimate; *p* = 0.909; no reference reported	Crude/unadjusted
* Zhao, 2015 *[26]	Poor-grade	Symptomatic CVS [unfavorable]	<24 h vs >24 h	4.3% (2/47)	11.3% (8/71)	No effect estimate; *p* = 0.312; no reference reported	Crude/unadjusted
*Non-significant numerical trends*							
* Lu, 2023 *[19]	Poor-grade	DCI	0–2 d (ref.) vs >10 d	32.1% (27/84)	57.1% (4/7)	aOR 2.74 (95% CI 0.49–15.49); *p* = 0.253; trend favoring earlier	Multivariable-adjusted

Dashes indicate unreported timing-specific proportions. [Unfavorable] denotes outcomes for which higher values are worse. Analysis type describes the displayed result, not all analyses in the source study. “Descriptive” indicates no formal comparative effect estimate; “crude/unadjusted,” no confounder adjustment. Reference categories are marked “(ref.)” in the Time window column when reported or identifiable from the model. No single categorical reference applies to descriptive comparisons, omnibus tests, comparisons lacking a categorical effect estimate, or continuous-time models. ORs from different models or outcome definitions are not directly comparable. Şimsek and Akinci [21]: the omnibus test included all four timing/treatment groups; no pairwise comparison was reported. Hostettler et al. [14]: the earlier-treatment association was restricted to HH grades 1–3 and strongest in HH grade 3. Kurisu et al. [16]: postoperative complication analyses excluded 21 patients who died early. *aOR* adjusted odds ratio, *CVS* cerebral vasospasm, *DCI* delayed cerebral ischemia, *DIND* delayed ischemic neurological deficit, *HH* Hunt–Hess grade, *OR* odds ratio, *PA* propensity-adjusted, *RCS* restricted cubic spline

Three studies reported findings compatible with earlier-treatment benefit. Şimsek and Akinci [21] found vasospasm rates of 5.9%, 25.7%, 11.1%, and 6.9% across the <6 h, 6–72 h, >72 h, and no-repair groups (omnibus *p* = 0.018), without a pairwise <6 h versus 6–72 h comparison. Lu et al. [19] found higher adjusted DCI risk with treatment on days 3–10 than days 0–2 (aOR 2.44). Treatment after day 10 was classified separately as a non-significant numerical trend in the same direction (aOR 2.74). Hostettler et al. [14] associated treatment >24 h with cerebral infarction in HH grades 1–3 (aOR 1.85), particularly HH grade 3 (aOR 7.69).

Vergouwen et al. [9] reported a hypothesis-generating descriptive finding favoring later treatment: DCI was numerically higher at <6 h than 6–24 h (32.7% [36/110] vs 26.1% [101/387]), without formal comparison.

Six studies reported null overall or outcome-specific findings. Buscot et al. [12] and Wang B. et al. [8] found no adjusted continuous-time association with DCI or postoperative cerebral ischemia, respectively. Hostettler et al. [14] found similar crude CVS rates and no adjusted overall association with cerebral infarction, despite the subgroup findings above. Koester et al. [15] reported similar crude vasospasm and no association with DCI-related infarction (PA-adjusted OR 0.87). Kurisu et al. [16] found similar descriptive DIND rates and no univariable association with delayed angiographic vasospasm. Zhao et al. [26] found no significant differences in symptomatic vasospasm (4.3% vs 11.3%) or cerebral infarction (19.1% vs 18.3%).

The evidence does not support a consistent association between earlier treatment and reduced vasospasm or related cerebral ischemia. Heterogeneous outcome definitions and predominantly unadjusted analyses limit this conclusion.

*Hydrocephalus.* Twelve studies reported hydrocephalus or shunt-dependent hydrocephalus; seven provided timing-based outcome analyses (Table 6): one favored later treatment, five found no difference, and one showed a non-significant numerical trend favoring later treatment. The remaining five reported baseline hydrocephalus or global complication rates without timing-based outcome analyses [10, 11, 18–20].

**Table 6:** Association between onset-to-treatment time and hydrocephalus or shunt-dependent hydrocephalus

Study	Population	Scale & time window	Early % (n/N)	Late % (n/N)	Effect measure (95% CI)	Analysis type
*Favoring later treatment*						
* Hostettler, 2023 *[14]	All grades	VPS, <24 h vs >24 h	27.5% (192/698)	12.3% (19/155)	No effect estimate; *p* < 0.001	Crude/unadjusted
*No difference*						
* Buscot, 2022 *[12]	All grades	Hydrocephalus, continuous (RCS)	–	–	Adjusted OR 1.00 per hour (95% CI 1.00–1.01); nonlinearity *p* = 0.430	Multivariable-adjusted RCS
* Kurisu, 2023 *[16]	All grades	Delayed hydrocephalus, ≤24 h vs >24 h	34.4% (56/163)	41.9% (13/31)	No formal comparison or effect estimate	Descriptive
* Şimsek, 2022 *[21]	Poor-grade	VPS (6 mo), <6 h vs ≥6 h or no repair	–	–	No effect estimate; *p* > 0.05	Crude/unadjusted
* Wu, 2022 *[23]	Poor-grade	Postoperative hydrocephalus, <24 h vs >24 h	48.4% (30/62)	45.8% (11/24)	No effect estimate; *p* = 0.832	Crude/unadjusted
* Zhao, 2015 *[26]	Poor-grade	Postoperative hydrocephalus, <24 h vs >24 h	8.5% (4/47)	9.9% (7/71)	No effect estimate; *p* = 1.000	Crude/unadjusted
*Non-significant numerical trends*						
* Wang B., 2026 *[8]	All grades	Postoperative hydrocephalus, continuous	–	–	aOR 0.991 per hour (95% CI 0.982–1.001); *p* = 0.080; trend favoring later treatment	Multivariable-adjusted continuous-time model

Dashes indicate unreported timing-specific proportions. *aOR* adjusted odds ratio, *RCS* restricted cubic splines, *VPS* ventriculoperitoneal shunt. Kurisu et al. [16]: postoperative complication analyses excluded 21 patients who died early. “Descriptive” denotes hypothesis-generating findings without a formal comparative effect estimate; findings based on few events warrant similar caution

Hostettler et al. [14] reported higher unadjusted shunt-dependent hydrocephalus with treatment <24 h than >24 h (27.5% vs 12.3%; *p* < 0.001). Because the analysis did not adjust for baseline severity, this finding may reflect confounding by indication, with more severely affected patients treated earlier.

Şimsek and Akinci [21] found no significant difference in shunt requirement between clipping within 6 h and the pooled ≥6 h/no-repair group (*p* > 0.05), but did not report timing-stratified counts. Buscot et al. [12] found no significant adjusted continuous-time association with hydrocephalus (aOR 1.00 per hour). Kurisu et al. [16] reported descriptively similar delayed-hydrocephalus rates at ≤24 h and >24 h (34.4% vs 41.9%), without formal comparison; this finding was considered hypothesis-generating. Wu et al. [23] and Zhao et al. [26] likewise found no significant timing-based differences. Wang B. et al. [8] reported only a non-significant numerical trend toward lower odds of postoperative hydrocephalus with increasing time (aOR 0.991 per hour).

Lu et al. [19] reported preoperative hydrocephalus across the 0–2-, 3–10-, and >10-day groups (57.1%, 38.9%, and 57.1%), and Ohbuchi et al. [20] reported acute hydrocephalus at admission in 43%; as baseline rather than post-treatment outcomes, these data were excluded from the timing-hydrocephalus outcome synthesis. In Ohbuchi, among patients treated within 72 h, baseline acute hydrocephalus was associated with longer treatment-completion time (20.6 vs 13.7 h; adjusted *p* = 0.0054). Loconi-Vallejos et al. [18] reported only a global rate, whereas Brawanski et al. [11] and Teo et al. [10] reported hydrocephalus as a complication without timing-stratified analyses.

Overall, the evidence does not support a consistent independent association between treatment timing and hydrocephalus. Baseline hemorrhage severity and clinical condition remain important potential confounders.

*Length of stay (LOS).* Five studies reported LOS; three provided timing-based analyses for comparative synthesis (Table 7), whereas two [10, 11] did not.

**Table 7:** Association between onset-to-treatment time and length of stay (LOS)

Study	Population	Scale & time window	Earlier timing group(s): LOS, d (*n*)	Later timing group: LOS, d (*n*)	Effect measure (95% CI)	Analysis type
*Favoring earlier treatment*						
* Tykocki, 2017 *[22]	Poor-grade (WFNS IV–V)	Neurosurgical-unit LOS (days), <24 h vs. >24 h (ref.)	–	–	Regression coefficient *B* = −0.65 d (95% CI not reported); *p* = 0.025	Multivariable-adjusted
* Wang B., 2025 *[7]	Poor-grade (WFNS IV–V)	Hospital LOS, median (IQR), <12 h vs. 12–23 h vs. ≥24 h	<12 h: 16.5 (IQR 7–26), *n* = 320; 12–23 h: 17.0 (IQR 11–24), *n* = 220	≥24 h: 20.0 (IQR 12–28), *n* = 450	No formal statistical comparison or effect estimate reported; descriptive trend favoring earlier treatment	Descriptive
*No difference*						
* Koester, 2023 *[15]	All grades	Hospital LOS (days), ≤24 h vs. >24 h (ref.)	19 ± 9 d (*n* = 94)	19 ± 9 d (*n* = 919)	Regression coefficient *β* = 0.13 d (95% CI −1.85 to 2.12); *p* = 0.90	Propensity-adjusted

Dashes indicate unreported timing-specific LOS values. Analysis type describes the displayed result, not all source analyses. “Descriptive” denotes a hypothesis-generating finding without a formal comparative effect estimate; findings based on few events warrant similar caution. Reference categories are marked “(ref.)” in the Scale & time window column when reported or identifiable from the model; none applies to descriptive comparisons

Tykocki et al. [22] reported an adjusted association between treatment <24 h and neurosurgical-unit LOS (*p* = 0.025). The authors interpreted this as shorter LOS with earlier treatment, although the coefficient direction (*B* = 0.65) was internally inconsistent. Wang B. et al. [7] descriptively reported shorter median hospital stays with treatment <12 h than ≥24 h (16.5 vs 20.0 days), without formal comparison. This hypothesis-generating difference may reflect survivor bias because in-hospital mortality was higher at <12 h than ≥24 h (17.2% vs 2.9%).

Koester et al. [15] found identical mean LOS at ≤24 h and >24 h (19 ± 9 days) and no propensity-adjusted association (*β* = 0.13).

LOS remains underexplored, and the evidence is insufficient to support a consistent independent association between treatment timing and LOS.

*Treatment modality.* Of 20 studies, 12 evaluated both clipping and endovascular treatment, five were clipping-only, and three endovascular-only. Direct comparisons are summarized in Table 8. B and *β* = regression coefficients for LOS in days; IQR = interquartile range; LOS = length of stay. Wang B. et al. [7] did not formally compare LOS, and the descriptive trend may reflect survivor bias. Koester et al. [15] labeled the estimate as exp(β), but because the confidence interval included negative values, it is presented as β.

**Table 8:** Clipping versus coiling for overall outcomes (independent of treatment timing)

Study	Population	Outcome	Clipping % (n/N)	Coiling % (n/N)	Effect measure (95% CI)	Analysis type
*Lu, 2023 *[19]	Poor-grade	DCI	41.7% (25/60)	37.3% (25/67)	Treatment modality was not significantly associated with DCI in the adjusted analysis; the modality-specific OR and p value were not numerically reported	Multivariable-adjusted; numerical estimate unavailable
*Kurisu, 2023 *[16]	All grades (subacute)	mRS 0–2 (3 mo) [favorable]	70.6% (12/17)	78.6% (11/14)	70.6% vs 78.6%; *p* = 0.613	Crude/unadjusted
*Kurisu, 2023 *[16]	All grades (subacute)	Delayed angiographic vasospasm	76.5% (13/17)	21.4% (3/14)	Higher with clipping (*p* = 0.002); DIND did not differ significantly (*p* = 0.098)	Crude/unadjusted
*Teo, 2017 *[10]	All-grade	mRS 3–6 (6 mo) [unfavorable]	34.0% (85/250)	25.3% (126/499)	Reported aOR 0.963 for clipping vs coiling (ref.) (95% CI 0.666–1.391); *p* = 0.839	Multivariable-adjusted
*Teo, 2017 *[10]	Favorable grade	Confirmed DCI	21.1% (32/152)	10.5% (43/408)	Crude p = 0.001; PSM: 23.3% vs 18.9%, *p* = 0.272	Crude comparison; propensity-matched analysis
*Teo, 2017 *[10]	Favorable grade	Rebleeding	0.7% (1/152)	1.5% (6/408)	*p* = 0.441	Crude/unadjusted
*Teo, 2017 *[10]	Favorable grade	Hydrocephalus requiring EVD	9.9% (15/152)	14.0% (57/408)	*p* = 0.197	Crude/unadjusted
*Tykocki, 2017 *[22]	Poor-grade	mRS 0–3 (discharge) [favorable]	40.4% (19/47)	37.5% (12/32)	*p* = 0.142	Crude/unadjusted
*Tykocki, 2017 *[22]	Poor-grade	Mortality (discharge)	14.9% (7/47)	37.5% (12/32)	*p* = 0.021, recalculated from the published counts	Crude/unadjusted

Analysis type describes the displayed result, not all source analyses. “Crude/unadjusted” denotes no confounder adjustment. *DCI* delayed cerebral ischemia, *DIND* delayed ischemic neurological deficit, *EVD* external ventricular drain, *PSM* propensity score matching. Teo et al. [10]: denominators reflect patients with available 6-month outcomes. The reported regression coefficient (0.038) and aOR (0.963) were internally inconsistent; the aOR was retained. Tykocki et al. [22]: both modality comparisons were crude. Author-reported *p* values for mRS 0–3 and mortality were 0.142 and 0.078; recalculation from published counts using Pearson’s chi-square test yielded 0.794 and 0.021, respectively. Kurisu et al. [16]: published aggregate vasospasm counts were retained although they could not be reproduced from supplementary patient-level tables. Comparisons based on few events are hypothesis-generating

Teo et al. [10] reported higher crude 6-month poor-outcome rates after clipping than coiling, but no independent association after adjustment (aOR 0.963, 95% CI 0.666–1.391; *p* = 0.839). In the good-grade subgroup, confirmed DCI was more frequent after clipping in the crude comparison (21.1% vs 10.5%; *p* = 0.001), but not in a separate propensity-matched full-cohort analysis (23.3% vs 18.9%; *p* = 0.272). Rebleeding and EVD-requiring hydrocephalus did not differ.

Tykocki et al. [22] reported similar crude mRS 0–3 rates at discharge after clipping and endovascular treatment (40.4% vs 37.5%; author-reported *p* = 0.142; recalculated Pearson *p* = 0.794). Crude mortality was lower after clipping (14.9% vs 37.5%); author-reported *p* = 0.078; recalculated *p* = 0.021. Both unadjusted comparisons were hypothesis-generating given the limited sample and event counts.

Kurisu et al. [16] found similar 3-month functional outcomes after clipping and coiling in the subacute subgroup (70.6% vs 78.6%; *p* = 0.613), and modality was not independently associated with clinical outcome in the full cohort (aOR 2.06; p = 0.198). In the same subgroup, delayed angiographic vasospasm was more frequent after clipping (76.5% vs 21.4%; p = 0.002), whereas DIND did not differ. Lu et al. [19] reported similar overall DCI rates after clipping and endovascular treatment (41.7% vs 37.3%) and no independent association with DCI.

Direct comparisons were heterogeneous and outcome-specific, without a consistent independent advantage for either technique.

Treatment modality was associated with treatment timing in opposite directions across clinical settings. In Hostettler et al.’s [14] all-grade cohort, clipping independently predicted longer admission-to-treatment time (median 9.7 vs 5.5 h; coefficient 14.51; *p* = 0.003). Conversely, clipping occurred earlier in poor-grade cohorts with intracranial hemorrhage and mass effect, reflecting clinical urgency [15, 22].

Six studies evaluated timing-outcome associations within modality strata or formally tested interaction (Table 9) [7–9, 12, 15, 19]. Three studies formally tested interaction. Wang B. et al. [8] found a significant modality-timing interaction for mortality (*p* = 0.041): the U-shaped association was more pronounced in the microsurgical group and more gradual in the endovascular group. Buscot et al. [12] found no mortality interaction (*p* = 0.250), and Wang B. et al. [7] found none for mortality (p = 0.981) or functional outcomes (all *p* ≥ 0.05).

**Table 9:** Formal interaction tests and modality-stratified timing-outcome analyses

Outcome	Analysis/interaction result	Studies	Finding
*Functional outcome*	Not formally tested	Koester, 2023 [15]; Lu, 2023 [19]	Within-modality timing analyses did not establish different timing-outcome associations between techniques
	Not formally tested	Vergouwen, 2023 [9]	Treatment <6 h vs 6–24 h (ref.) was associated with higher poor-outcome risk in both endovascular (aRR 1.34, 95% CI 1.06–1.70) and microsurgical strata (aRR 1.45, 95% CI 1.01–2.08); no formal interaction test was reported
	Formal interaction not significant (all *p* for interaction ≥ 0.05)	Wang B., 2025 [7]	No significant modality-timing interaction was reported for the evaluated functional outcomes
*Mortality*	Not significant (*p* = 0.25)	Buscot, 2022 [12]	Technique did not modify the nonlinear timing-mortality association
	Not formally tested	Koester, 2023 [15]	Timing-mortality comparisons were null within both open microsurgical and endovascular strata
	Not significant (*p* = 0.981)	Wang B., 2025 [7]	Adjusted timing-mortality associations were similar in neurosurgical and endovascular strata
	Significant (*p* = 0.041)	Wang B., 2026 [8]	U-shaped timing-mortality relationship more pronounced in microsurgery group; endovascular group showed more gradual association
*Rebleeding/rerupture*	Not formally tested	Koester, 2023 [15]	Rerupture rates were similar across timing groups within both open microsurgical and endovascular strata
*Vasospasm/related cerebral ischemia*	Not formally tested	Lu, 2023 [19]	Timing associations with DCI were non-significant within both clipping and coiling strata; no formal interaction test was reported
*Length of stay*	Not formally tested	Koester, 2023 [15]	Length of stay did not differ by treatment timing within either open microsurgical or endovascular strata

Modality-stratified findings without formal interaction testing are hypothesis-generating and do not establish the presence or absence of effect modification. Only studies reporting an explicit modality × timing interaction were classified as formal interaction analyses. *aRR* adjusted risk ratio, *DCI* delayed cerebral ischemia

The other three studies reported stratified analyses without formal interaction testing. Vergouwen et al. [9] found higher adjusted poor-outcome risk with treatment <6 h in both modality strata. Koester et al. [15] found null timing comparisons within both strata across outcomes, and Lu et al. [19] found no clearly different timing associations for functional outcome or DCI. Only one mortality interaction was significant and was not reproduced; the hypothesis-generating stratified analyses showed no consistent modality-specific timing pattern.

Associations between onset-to-treatment time and aSAH outcomes were inconsistent. Earlier securement likely reduced pretreatment rebleeding, although treated-cohort findings varied; no clear association emerged for functional outcome, vasospasm, or hydrocephalus. Dichotomous mortality analyses conflicted, whereas two large continuous-time studies found nonlinear associations [8, 12]. Wang B. et al. [8] identified a significant U-shaped mortality pattern, with estimated risk lowest at approximately 32.6 h.

Direct clipping-coiling comparisons were heterogeneous, with no consistent independent association with clinical outcomes. Of six studies assessing timing-outcome associations by modality or formally testing interaction, only one found a significant modality-timing interaction; other formal and stratified analyses showed no reproducible modality-specific pattern. Nevertheless, modality-timing associations differed in direction by clinical severity and treatment selection.

Crude, descriptive, and subgroup-only findings were retained for completeness but not considered equivalent to analyses adjusted for baseline severity and major confounders.

## Discussion

Several systematic reviews and meta-analyses have examined the association between treatment timing and outcomes in aneurysmal subarachnoid hemorrhage (aSAH), but most have focused on narrow outcome sets or specific treatment modalities. de Gans et al. [27] and Yao et al. [28] analyzed composite unfavorable outcome according to surgical timing; Zhao et al. [29] focused on poor-grade patients; Rawal et al. [30] examined endovascular treatment specifically; and Ogolo et al. [31] evaluated surgical, endovascular, and pharmacological strategies across 3069 patients. The present review extends this evidence base by simultaneously examining the full spectrum of clinical outcomes – vasospasm, hydrocephalus, rebleeding, functional outcome, mortality, and length of stay – while assessing whether treatment modality modifies this relationship. Furthermore, by incorporating two large studies that modeled onset-to-treatment time as a continuous variable [8, 12], we were able to evaluate endpoint-specific continuous associations, including a potential U-shaped relationship between treatment timing and mortality, which may help reconcile discrepant findings in the literature.

The domain-based assessment identified high concern for selection into the timing analysis in every included report, most commonly because inclusion was conditioned on receipt of treatment and survival until aneurysm repair. Other recurrent limitations included incomplete adjustment for clinical and logistical determinants of timing, non-standardized timing ascertainment, and incomplete or variable follow-up. Accordingly, propensity-adjusted and more comprehensively adjusted analyses drove the interpretation, whereas crude or descriptive comparisons and adjusted models with serious concerns regarding model specification, post-exposure adjustment, or attrition were given lower interpretive weight. Descriptive findings, particularly those without formal comparisons or based on small event counts, were treated as hypothesis-generating. Residual confounding and survivor bias nevertheless remained possible even in the comparatively stronger analyses.

*Functional outcome and the challenge of confounding by indication.* The relationship between treatment timing and functional outcome has been investigated in several meta-analyses, with conflicting conclusions. Yao et al. [28], synthesizing 14 studies with over 14,000 patients, found that early surgery was associated with a lower risk of poor outcome in both good-grade (RR 0.65) and poor-grade (RR 0.71) patients, but acknowledged that age remained a residual confounder and that most included studies lacked robust adjustment for clinical severity. Zhao et al. [29], in 85 studies comprising 4506 poor-grade patients, reported higher rates of good neurological outcome with ultra-early treatment (< 48 h) (61% vs 40–47%), yet noted that nearly all included studies were retrospective and had a medium to high risk of bias, precluding adequate confounder adjustment.

Our review identified eight studies categorized as favoring earlier treatment, four favoring later treatment, four finding no independent association, and three reporting non-significant numerical trends. However, these findings ranged from descriptive and unadjusted comparisons to adjusted analyses and should not be interpreted as methodologically equivalent, challenging a causal interpretation. Large-scale evidence from the Nationwide Inpatient Sample showed that admission-to-treatment delays >3 days were associated with higher adjusted odds of moderate-to-severe neurological disability than treatment within 3 days (OR 1.29; 95% CI 1.16–1.43; *p* < 0.0001) [32]. However, the database lacked Hunt and Hess grade, vasospasm, and rerupture data and did not capture time before transfer.

The most consistent explanation for discrepancies across early-treatment windows is confounding by indication. Patients with greater clinical severity are routinely treated more urgently, which can create a spurious association between early intervention and worse prognosis in unadjusted analyses. This pattern was evident in several cohorts with baseline severity imbalances, including Kurisu et al. [16], in which patients treated after 24 h had milder clinical severity at presentation. Crude associations often attenuated after multivariable or propensity-based adjustment [10, 14, 15, 26]. Vergouwen et al. [9] showed a different pattern in the primary analysis, in which treatment <6 h remained associated with a higher risk of poor outcome after adjustment (aRR 1.36; 95% CI 1.11–1.66). However, this association attenuated after reclassification of patients with early rebleeding (aRR 1.03; 95% CI 0.82–1.29) and after exclusion of all patients with rebleeding (aRR 1.23; 95% CI 0.96–1.58), underscoring the likely influence of confounding by indication. Because treatment timing was not randomized, residual confounding by clinical severity and clinician-driven treatment selection cannot be excluded. In a meta-analysis of 3287 poor-grade patients, de Winkel et al. [33] identified age, clinical grade, pupillary light reflex, and modified Fisher grade as the primary independent predictors of functional outcome but did not find consistent evidence that treatment timing was an independent predictor.

Chuck et al. [34], analyzing 351 endovascularly treated patients, found that longer arrival-to-treatment time was paradoxically associated with better outcomes among high-grade patients (OR 0.54; *p* = 0.024), a finding the authors attributed to clinician-driven triage rather than a causal benefit of delay.

Three studies in our review reported paradoxical findings that challenge a linear benefit model. In addition to Vergouwen et al. [9], Wang B. et al. [7] reported lower adjusted odds of unfavorable outcome with treatment ≥24 h than with treatment <12 h. Wang B. et al. [8] found that longer onset-to-treatment time was associated with lower odds of 2-year dependent survival. Continuous modeling showed a monotonically decreasing association, with the risk declining most rapidly during the first 12 h and reaching a plateau at approximately 32.6 h. This functional-outcome pattern was not U-shaped and should not be interpreted as evidence that treatment delay improves functional recovery, because residual confounding by clinical severity remains likely.

Overall, the available evidence does not permit a causal conclusion that ultra-early treatment either improves or worsens functional outcome. Adjustment often attenuated crude timing associations, but this was not uniform; multivariable and propensity-adjusted analyses also produced heterogeneous results. Associations favoring earlier treatment were reported most often in poor-grade cohorts; however, these findings remained vulnerable to treated-survivor selection and residual confounding and were not considered more credible solely because the cohorts were restricted to poor-grade patients. Across both poor-grade and all-grade cohorts, baseline hemorrhage severity and clinical condition were more consistently associated with functional outcome than treatment timing.

*Mortality and the U-shaped relationship.* Across the 12 studies in our review that provided timing-based mortality analyses, the overall evidence was conflicting. Two studies reported lower mortality with earlier treatment, three reported higher mortality with earlier treatment, three showed non-significant numerical trends, and four found no difference. These discordant results are likely influenced by opposing selection mechanisms. Patients with greater clinical severity are often treated more rapidly, whereas patients treated later must survive long enough to enter the delayed-treatment group. These mechanisms may directly distort the observed shape of the time-outcome association and cannot be fully corrected through multivariable adjustment or propensity-based methods in retrospective analyses.

Two large multicenter studies modeled onset-to-treatment time continuously using cubic spline models. Buscot et al. [12] observed a mortality nadir at approximately 12.2 h, but the nonlinear association was not statistically significant after adjustment (*p* = 0.12). Wang B. et al. [8], in the largest cohort in this review (*n* = 3560), reported a significant U-shaped relationship with a mortality nadir at approximately 32.6 h. Both findings remain observational and susceptible to confounding by indication and survivor bias. Together, they raise the hypothesis that a nonlinear association may help reconcile seemingly contradictory findings in the broader literature. Systematic reviews and meta-analyses based on dichotomized time windows have also reached divergent conclusions. Yao et al. [28] found that early surgery was associated with a lower risk of poor outcome than delayed surgery. Zhao et al. [29] reported that ultra-early treatment (< 48 h) was associated with the highest rates of good neurological outcome and the lowest mortality. Conversely, Rawal et al. [30] described inconsistent results for early treatment within 1 day.

The divergence across these reviews may partly reflect the limitations of dichotomous time cutoffs. Studies comparing “<24 h vs >24 h” or “<48 h vs >48 h” combine patients treated at substantially different times within the same category, potentially masking associations that cannot be adequately represented by a single cutoff. Although both continuous-time studies suggested nonlinear patterns, only Wang B. et al. [8] reported a statistically significant adjusted nonlinear association, and their estimated nadirs differed considerably (approximately 12.2 h vs 32.6 h). This discrepancy may partly reflect differences in study populations and inclusion criteria. Buscot et al. [12] included treated patients without restricting inclusion to treatment within 72 h, whereas Wang B. et al. [8] included only patients treated within 72 h of onset. Both studies nevertheless conditioned inclusion on receipt of aneurysm repair, thereby excluding patients who died before treatment or remained untreated and who may have had greater baseline severity; Wang B. et al. additionally excluded patients treated after 72 h. These differences in cohort composition and the represented treatment-time range may have contributed to the distinct estimated nadirs. Conversely, patients selected for very early treatment are often more severely ill, introducing confounding by indication that may make earlier treatment appear comparatively unfavorable.

Differences in healthcare systems, patient mix, including the proportion of poor-grade patients, and mortality assessment periods (12-month mortality vs all-cause mortality during follow-up) may also have contributed. More generally, comparisons involving delayed-treatment groups are conditioned on survival until treatment, introducing survivor bias that may make later treatment appear comparatively favorable. Neither confounding by indication nor survivor bias can be fully corrected in retrospective analyses; therefore, the observed associations should not be interpreted as evidence that delaying treatment is beneficial. Future prospective studies with standardized inclusion criteria and continuous time modeling are needed to determine whether the association between treatment timing and mortality is reproducibly nonlinear and whether it varies according to patient subgroup (e.g., poor-grade vs good-grade, clipping vs coiling).

The distinct mortality nadirs reported at approximately 12.2 and 32.6 h should not be interpreted as defining a common therapeutic window. Rather, despite their substantial clinical and methodological differences, the two studies generate the hypothesis that the association between treatment timing and mortality may be nonlinear. The included studies did not directly evaluate the biological mechanisms underlying these observed associations; prospective confirmation is therefore required.

These hypothesis-generating findings do not conflict with the 2023 AHA/ASA Class 1 recommendation to treat “as early as feasible, preferably within 24 h of onset” [2]. The guideline’s language “as early as feasible” acknowledges that patient stabilization, diagnostic workup, and interhospital transfer may require time. However, the available observational evidence does not establish benefit for a specific 12–32-h interval or support a clinically actionable timing target within the first 24 h.

*Rebleeding.* The relationship between treatment timing and rebleeding risk in aSAH is conceptually straightforward – securing the aneurysm earlier should reduce the opportunity for pretreatment rebleeding – yet the published evidence appears contradictory. Our findings suggest that much of this contradiction is methodological rather than biological. Pretreatment rebleeding while the aneurysm remains unsecured and post-treatment rerupture are clinically and biologically distinct. Post-treatment rerupture reflects treatment durability and occlusion quality rather than the pretreatment interval. These outcomes were analyzed separately, and studies reporting mixed or unspecified events were not considered directly comparable. Reviews that conflate pre- and post-treatment rebleeding may underestimate the benefit of early intervention.

The clearest crude support for earlier aneurysm securement came from comparisons that included untreated or very late-treated patients [20, 21]. In Şimsek and Akinci [21], the untreated group had 49.1% rebleeding and 96.6% 6-month mortality; however, rebleeding timing was not specified, and the comparison was highly susceptible to treatment-selection and era bias. These findings align with classic data: Ohkuma et al. [35] reported 18.2% rebleeding before neurosurgical management within 6 h of ictus, and Park et al. [36] demonstrated that a formal 24/7 emergency protocol reduced in-hospital rebleeding from 7.4% to 2.1% (*p* = 0.003). Recent meta-analyses reinforce the direction of effect: Munasinghe et al. [4] found that ultra-early treatment (≤ 6 h) reduced rebleeding (OR 0.21; 95% CI 0.06–0.77), and Ogolo et al. [31] reported a pooled OR of 0.57 (95% CI 0.39–0.82) favoring early intervention.

The timing-stratified analysis of Brawanski et al. [11] included only treated patients with a reliably documented time of ictus and excluded untreated patients. The study did not specify whether rebleeding occurred before or after aneurysm repair. Survivor bias or selective treatment allocation may therefore explain the progressively lower crude rebleeding proportions observed in the later-treatment groups. In the absence of an adjusted analysis, these data do not directly demonstrate a protective effect of earlier treatment and should not be interpreted as evidence of a causal benefit from delay. Across the remaining treated cohorts, findings were null, descriptive, or based on few events [7, 12, 14, 15, 17, 22, 26]. Wang B. et al. [8] reported a small inverse association between treatment delay and postoperative rebleeding, but this isolated finding was not reproduced and concerns post-treatment durability rather than the pretreatment interval.

These null or paradoxical findings remain vulnerable to the selection mechanisms described above and therefore require study-specific interpretation. In Brawanski et al. [11], inclusion was restricted to treated patients with reliable ictus timing, excluding untreated patients and those without complete timing data; the progressively lower crude rebleeding proportions in later groups therefore cannot be interpreted causally. In Vergouwen et al. [9], patients treated within 6 h had greater baseline severity and a higher crude proportion of “any rebleeding” than those treated at 6–24 h (26.4% vs 12.7%). However, pre- and post-treatment events were not separated, and no adjusted rebleeding analysis was reported. Wang B. et al. [8] reported a small inverse association for postoperative rebleeding, but this isolated finding was not reproduced and concerns treatment durability rather than the pretreatment interval. Prior literature likewise identifies selection bias and incomplete confounder adjustment as major explanations for inconsistent timing findings [15, 28, 30, 38].

The potential impact of treatment delay on rebleeding is illustrated by the ISAT post hoc analysis of van Donkelaar et al. [39]. The interval between randomization and treatment was significantly longer for patients allocated to clipping (1.7 vs 1.1 days; p < 0.0001). During this delay, 17 patients died from rebleeding in the clipping arm compared with only 6 in the coiling arm (RR 2.81; 95% CI 1.11–7.11). The median interval between randomization and fatal rebleeding was 2 days, and all pretreatment deaths occurred in UK centers, where time to treatment was longer than in non-UK centers (mean 1.4 vs 0.4 days). After these pretreatment deaths were excluded, the 5-year difference between clipping and coiling was no longer significant, suggesting that delay-related rebleeding contributed to the early ISAT advantage for coiling.

These same confounders have been identified across the broader literature as key sources of inconsistency in timing studies. Rawal et al. [30] described the results of early endovascular treatment within 24 h as “inconsistent” and attributed the variability to between-study differences in confounder adjustment. Yao et al. [28], in a meta-analysis of 14 studies, explicitly acknowledged that most included studies lacked robust adjustment for clinical severity.

Our findings are consistent with the AHA/ASA guideline [2], which recommends treatment as early as feasible (Class I, Level B-NR) based on rebleeding prevention. The NICE guideline [40] found insufficient evidence for a specific threshold, reflecting the uncertainty generated by the methodological issues identified here.

Earlier aneurysm securement is likely to reduce pretreatment rebleeding, particularly when untreated or markedly delayed patients are included, whereas observational comparisons among treated patients remain inconsistent and highly susceptible to selection bias.

*Vasospasm/related cerebral ischemia, hydrocephalus, and length of stay: outcomes without a consistent timing signal.* In contrast to pretreatment rebleeding, vasospasm, delayed cerebral ischemia (DCI), other cerebral ischemic complications, hydrocephalus, and length of stay (LOS) showed no reproducible independent association with treatment timing across overall study populations, although isolated adjusted and subgroup findings were reported.

Thirteen studies reported vasospasm or related cerebral ischemic complications, of which nine provided usable timing-based analyses. Both continuous-time analyses were null: Buscot et al. [12] found no association between onset-to-treatment time and DCI (adjusted OR 1.00 per hour; p for nonlinearity = 0.88), and Wang B. et al. [8] found no association with postoperative cerebral ischemia (adjusted OR 1.001 per hour; *p* = 0.753). Among categorical analyses, Şimsek and Akinci [21] reported lower unadjusted vasospasm rates with treatment <6 h, whereas Lu et al. [19] found higher adjusted odds of DCI among poor-grade patients treated at 3–10 days rather than 0–2 days (aOR 2.44; 95% CI 1.06–5.64; *p* = 0.036); the estimate for treatment >10 days was imprecise and not significant. Conversely, the higher DCI proportion reported with treatment <6 h by Vergouwen et al. [9] was descriptive and lacked a formal comparison, while propensity-adjusted findings from Koester et al. [15] and analyses from Kurisu et al. [16] and Zhao et al. [26] showed no significant timing association.

Hostettler et al. [14] similarly found no significant overall association with cerebral infarction (aOR 1.56; 95% CI 0.92–2.70; *p* = 0.10), but reported higher risk with treatment >24 h among patients with HH grades 1–3 (aOR 1.85; 95% CI 1.01–3.33; *p* = 0.05), particularly those with HH grade 3 (aOR 7.69; 95% CI 2.44–25.00; *p* < 0.001). Thus, although selected analyses suggested higher ischemic risk with either earlier or later treatment, these signals were not reproduced across overall populations and frequently arose from subgroup, descriptive, or unadjusted comparisons.

A distinct timing-specific finding was reported by Santos et al. [41], who found that treatment during the vasospasm phase (days 4–14 post-ictus) was independently associated with a higher risk of cerebral infarction distal to the treated vessel (aOR 2.02; *p* < 0.01) and poor outcome (aOR 2.05). This finding suggests that treatment during the vasospasm phase may be associated with greater procedural risk. However, it does not establish that procedural complications explain the potential U-shaped mortality association observed in the continuous-time studies, because that relationship and its underlying mechanisms were not directly evaluated.

For hydrocephalus, 12 studies reported hydrocephalus or shunt-dependent hydrocephalus, but only seven provided usable timing-based analyses. Continuous-time models from Buscot et al. [12] and Wang B. et al. [8] showed no significant associations, and the categorical analyses from Şimsek and Akinci [21], Kurisu et al. [16], Wu et al. [23], and Zhao et al. [26] were also null or showed only non-significant numerical differences. The principal exception was Hostettler et al. [14], in which shunt-dependent hydrocephalus was more frequent among patients treated <24 h than among those treated later (27.5% vs 12.3%; *p* < 0.001). Because this comparison was unadjusted and patients with greater clinical severity were treated earlier, the apparent protective effect of delayed treatment may reflect confounding by indication. Overall, the available evidence does not support an independent association between treatment timing and hydrocephalus and is more consistent with an important role for initial hemorrhage severity.

Treatment modality may nevertheless contribute to hydrocephalus risk. In a propensity score-matched analysis, Koyanagi et al. [42] identified a higher incidence of shunt-dependent hydrocephalus after surgical clipping than after endovascular coiling (30% vs 16%; OR 2.2; p = 0.009). This suggests that treatment technique may be an important source of heterogeneity in post-aSAH hydrocephalus risk, independent of the inconsistent evidence regarding onset-to-treatment time.

LOS remains underexplored as a formal outcome. Five studies reported LOS, but only three provided usable timing-based data. Tykocki et al. [22] found that treatment within 24 h independently predicted shorter neurosurgical-unit LOS among poor-grade patients (adjusted *B* = − 0.65; *p* = 0.025). In contrast, Koester et al. [15] reported identical mean hospital stays in the ≤24-h and >24-h groups (19 ± 9 days in both) and no association after propensity adjustment (*β* = 0.13 days; 95% CI −1.85 to 2.12; *p* = 0.90). Wang B. et al. [7] descriptively reported shorter median LOS with treatment <12 h than with treatment ≥24 h (16.5 vs 20.0 days), but no formal comparison was performed, and the numerical pattern may have been affected by survivor bias because in-hospital mortality was higher in the <12-h group than in the ≥24-h group (17.2% vs 2.9%). These limited and discordant findings are insufficient to support a consistent independent association between treatment timing and LOS.

The inconsistency across these secondary outcomes is consistent with the between-center and between-country variability documented in the SAHIT repository [43], which found that center-level factors explained outcome differences not attributable to patient characteristics or treatment timing. This variability further cautions against attributing vasospasm-related complications, hydrocephalus, or LOS to the onset-to-treatment interval alone.

*Treatment modality considerations.* Direct clipping-versus-coiling comparisons within the studies included in this review were heterogeneous and outcome-specific. Teo et al. [10] reported a higher crude 6-month poor-outcome rate after clipping than after coiling (34.0% vs 25.3%), but treatment modality was not independently associated with outcome after multivariable adjustment (aOR 0.963; 95% CI 0.666–1.391; *p* = 0.839). The higher crude rate of confirmed DCI after clipping in the good-grade subgroup (21.1% vs 10.5%; *p* = 0.001) was not reproduced in a separate propensity-matched full-cohort analysis (23.3% vs 18.9%; *p* = 0.272), and rebleeding and EVD-requiring hydrocephalus did not differ significantly. In the poor-grade cohort of Tykocki et al. [22], crude favorable functional-outcome rates were similar after clipping and endovascular treatment (40.4% vs 37.5%); the lower crude mortality after clipping (14.9% vs 37.5%) was based on few events and lacked confounder adjustment and was therefore considered hypothesis-generating. Kurisu et al. [16] found similar 3-month functional outcomes after clipping and coiling in the subacute subgroup and no independent association between modality and clinical outcome in the full cohort. Although delayed angiographic vasospasm was more frequent after clipping in that subgroup, DIND did not differ significantly. Lu et al. [19] similarly found no independent association between treatment modality and DCI. Taken together, the included studies did not establish a consistent independent advantage of either modality.

The broader literature further demonstrates that comparative findings vary according to clinical severity, patient selection, and study design. In ISAT [44], 2143 patients with ruptured aneurysms considered suitable for either technique were randomized to clipping or coiling. Endovascular coiling reduced the proportion of patients dead or dependent at 1 year (23.7% vs 30.6%; p = 0.0019), corresponding to an absolute risk reduction of 6.9%. Shao et al. [45], in a meta-analysis of five RCTs comprising 2,883 patients, likewise found a higher risk of poor functional outcome with clipping (RR 1.34; 95% CI 1.18–1.51), but no significant mortality difference. Li et al. [46] found that the functional benefit of coiling was evident in RCTs but not statistically significant in observational studies. In their subgroup analysis of 437 poor-grade patients, poor-outcome rates were similar after clipping and coiling (42.6% vs 43.0%; OR 0.88; 95% CI 0.56–1.38; *p* = 0.570). Thus, the available subgroup evidence did not demonstrate the functional advantage observed in broader RCT populations, although this null finding should not be interpreted as proof of equivalence.

The ISAT post hoc analysis by van Donkelaar et al. [39] suggested that pretreatment rebleeding deaths during the longer interval between randomization and clipping contributed to the early difference between the treatment groups (RR 2.81). After these pretreatment deaths were excluded, the 5-year difference was no longer statistically significant. This finding supports a contribution from delay-related rebleeding but does not establish equivalence between the techniques or demonstrate that their outcomes would necessarily be identical if treatment were delivered at the same time. In a contemporary National Inpatient Sample analysis of 6555 clipping and 15,350 coiling hospitalizations, Deutsch et al. [47] found no adjusted differences in overall complications, neurological complications, in-hospital mortality, or total charges. These findings further illustrate the convergence of risk-adjusted outcomes in selected contemporary populations without establishing general equivalence between modalities.

Treatment modality was also associated with treatment timing in different directions across clinical settings. In the all-grade cohort of Hostettler et al. [14], clipping independently predicted a longer admission-to-treatment interval (median 9.7 vs 5.5 h; regression coefficient 14.51; *p* = 0.003). Conversely, clipping occurred earlier in poor-grade cohorts, particularly among patients with intracranial hemorrhage or mass effect [15, 22], a pattern consistent with urgency-driven treatment selection. Attenello et al. [32] found that younger age and treatment at a teaching hospital were associated with lower odds of a clipping delay >3 days but were not significantly associated with time to coiling, indicating that the determinants of admission-to-procedure delay differed by modality in that database.

Surgeon experience was also associated with treatment timing. Elbir et al. [48] found that greater neurosurgical experience was independently associated with delayed surgery. Early surgery was performed in 5.8% of patients treated by surgeons with >200 aneurysm procedures, compared with 42.6% and 63.6% of those treated by surgeons with 101–200 and ≤100 procedures, respectively (OR 19.199; 95% CI 5.881–62.682; *p* < 0.001). The authors suggested that differences in emergency-shift participation, adherence to traditional delayed-surgery teaching among older surgeons, and the greater willingness of younger surgeons to operate outside regular working hours may have contributed to this association; however, these explanations were not directly tested.

Regarding effect modification, six studies evaluated timing-outcome associations within modality strata or formally tested modality-timing interaction [7–9, 12, 15, 19]. Three performed formal interaction testing. Wang B. et al. [8] identified a significant interaction for mortality (*p* = 0.041), with a more pronounced U-shaped association in the microsurgical group and a more gradual association in the endovascular group. Although Ogolo et al. [31] suggested a broadly similar modality-specific pattern, this did not constitute a formal replication of the interaction. Buscot et al. [12] found no significant modality-timing interaction for mortality (*p* = 0.250), and Wang B. et al. [7] found no interaction for mortality (*p* = 0.981) or the evaluated functional outcomes (all *p* ≥ 0.05).

The remaining three studies reported modality-stratified analyses without formal interaction testing. Vergouwen et al. [9] found a higher adjusted risk of poor outcome with treatment <6 h in both endovascular and microsurgical strata. Koester et al. [15] reported null timing comparisons within both modalities across the evaluated outcomes, and Lu et al. [19] did not identify clearly different timing associations for functional outcome or DCI. Because within-modality analyses without a formal interaction test cannot establish either the presence or absence of effect modification, these findings remain hypothesis-generating. Overall, whether treatment modality modifies the timing-outcome association remains uncertain: only one of three formal analyses identified a significant interaction, and that finding was not reproduced. Current evidence is therefore insufficient to support different treatment-timing targets for clipping and endovascular treatment.

*Methodological aspects and limitations.* This review has several limitations. First, two reports were derived from overlapping populations in the same multicenter database, reducing the effective number of independent participants and potentially overrepresenting that data source in the narrative synthesis. Eighteen of the 20 included studies were retrospective cohort studies, and the remaining two were post hoc analyses of randomized trial data [9, 10]. Because treatment timing was not randomized in either post hoc analysis, all included studies were observational with respect to the exposure of interest, and no randomized evidence for the timing-outcome association was available. These observational comparisons are susceptible to selection bias – including confounding by indication and survivor bias – and to residual confounding because covariate adjustment varied across studies. These mechanisms may distort the direction, magnitude, and shape of the observed timing-outcome association and limit causal inference. This weakness characterizes the broader evidence base: Zhao et al. [29] reported that the majority of the 85 studies included in their meta-analysis had a medium to high risk of bias, while de Winkel et al. [33] noted that most included studies had small sample sizes and a high risk of bias.

Second, substantial heterogeneity in the definitions and categorization of treatment timing precluded quantitative synthesis. Consequently, this review could not estimate a pooled effect or determine whether any specific time cutoff was consistently associated with outcome. This problem is pervasive in the literature. De Winkel et al. [33] noted that non-uniform definitions of favorable outcome and variable categorization of treatment windows complicated interpretation, while Yao et al. [28] similarly reported that inconsistent definitions of early and late surgery complicated data synthesis.

Third, publication bias and selective reporting may have affected the available evidence. Because quantitative pooling was not performed and relatively few studies contributed to each specific timing-outcome comparison, publication bias could not be formally assessed in the present review. Previous meta-analyses have examined this issue using different approaches: Yao et al. [28] applied Egger’s and Begg’s tests, while Zhao et al. [29] acknowledged that centers with better outcomes may be more likely to report their results.

Fourth, the predominance of studies from high-volume or highly specialized centers in Asia and Europe limits generalizability. The evidence was generated predominantly in settings with comparatively greater access to timely neuroimaging, neurocritical care, and both surgical and endovascular resources. These conditions may differ substantially from those in low- and middle-income countries. In Brazil, Gonçalves et al. [49] prospectively followed 471 patients with aSAH at two high-volume referral centers and found a median onset-to-arrival interval of 4 days, substantially longer than the early-treatment windows that predominated in this review. Only 25% of patients underwent endovascular coiling, and in-hospital mortality was 24%. Similarly, Souza et al. [50], in a São Paulo cohort of 212 patients, reported a median ictus-to-treatment interval of 9 days, with 15.6% of patients receiving no definitive treatment. In both cohorts, poor clinical grade remained the dominant predictor of unfavorable outcome, consistent with our findings. However, the prolonged delays and limitations in access to definitive and endovascular treatment represent system-level barriers that are not captured by comparisons among treated cohorts and cannot be overcome by defining a treatment-time cutoff alone. The between-center variability documented by Dijkland et al. [43] in the SAHIT repository (median odds ratio 1.26; 95% CI 1.16–1.52) further indicates that outcomes vary meaningfully across institutions. Findings from high-volume specialized centers may therefore not extrapolate to lower-volume, resource-constrained, or referral-delay settings.

Finally, interrater reliability for quality assessment was not formally evaluated, preventing quantification of agreement between reviewers. In addition, although AI assistance was used during title screening, this approach requires further methodological validation in systematic reviews. These limitations should be considered when interpreting the reproducibility and comprehensiveness of the study-selection and quality-assessment processes.

*Implications for clinical practice and future research.* The certainty of evidence for the association between treatment timing and mortality was very low because of serious risk of bias, inconsistency, and imprecision. Accordingly, the findings of this review do not support defining an optimal treatment window or recommending any specific treatment interval to reduce mortality. Two studies that modeled treatment time as a continuous exposure raise the hypothesis that the timing-mortality association may be nonlinear. However, their distinct estimated nadirs – approximately 12.2 and 32.6 h – and substantial clinical and methodological differences preclude interpreting either estimate as a validated or clinically actionable treatment target or combining them into a 12–32-h therapeutic window. The apparent nonlinear associations may also be influenced by confounding by indication, because more severely ill patients are often treated earlier, and by survivor bias, because patients treated later must survive until treatment. These biases cannot be fully corrected in retrospective analyses. The need for further investigation of treatment timing as a continuous exposure is also reflected in contemporary literature: Chuck et al. [34] concluded that modest variation in arrival-to-treatment time within the AHA-recommended 24-h period did not support ultra-early aneurysm securement, particularly among high-grade patients, and called for larger prospective multicenter studies. The available evidence should not be interpreted as demonstrating that treatment delay is beneficial or as providing a basis for modifying current guideline recommendations.

Future research should model treatment time as a continuous exposure using flexible methods such as restricted cubic splines, rigorously adjust for clinical severity and age, and stratify analyses by treatment modality, with formal interaction testing where appropriate. As Yao et al. [28] emphasized, the effect of ultra-early surgery remains unresolved. Prospective registries with standardized timing and outcome definitions are urgently needed to enable meaningful cross-study comparisons and meta-analytic synthesis. The association between treatment timing and pretreatment rebleeding also warrants further investigation. Finally, collaborative research networks across high- and middle-income countries are needed to determine whether the hypothesized nonlinear time-outcome association can be reproduced in settings where delayed presentation is common, using study designs and analyses that better address confounding by indication and survivor bias.

## Conclusion

This systematic review of 20 reports comprising 11,096 participant records suggests that the relationship between onset-to-treatment time and clinical outcomes is not uniform across the outcomes examined. Earlier aneurysm securement remains supported for the prevention of pretreatment rebleeding, although comparisons restricted to treated cohorts were inconsistent. In contrast, the independent association between treatment timing and mortality or functional outcome remains uncertain because of severe confounding by indication, survivor bias, and heterogeneous time definitions. The certainty of evidence for the mortality association was very low because of serious risk of bias, inconsistency, and imprecision. Vasospasm-related ischemia, hydrocephalus, and length of stay likewise showed no reproducible independent association with treatment timing.

Two observational studies using continuous-time modeling raise the hypothesis that the timing-mortality association may be nonlinear. However, their distinct estimated nadirs at approximately 12.2 and 32.6 h, substantial clinical and methodological differences, and susceptibility to confounding preclude interpreting either estimate – or the interval between them – as a validated or clinically actionable therapeutic window. Current guideline recommendations for aneurysm securement as early as feasible, ideally within 24 h, remain supported by the available rebleeding data. The findings do not support intentional treatment delay, a specific alternative timing target, or modification of these recommendations. Evidence regarding modality-timing interaction remains limited and inconsistent. Because the evidence was generated predominantly in high-income healthcare settings, its applicability to settings with delayed presentation and limited access to endovascular treatment remains uncertain. Prospective multicenter registries using standardized timing and outcome definitions, continuous-time modeling, and rigorous confounder adjustment are needed to determine whether the hypothesized nonlinear association is reproducible across diverse healthcare settings.

## Supplementary Information

Below is the link to the electronic supplementary material.

Supplementary file1 (DOCX 31 KB)

## Data Availability

This systematic review used only previously published data; therefore, ethical approval was not required. Consent to participate and consent for publication are not applicable. All data generated or analysed during this study are included in this published article and its supplementary information files; code availability is not applicable. This systematic review was conducted in accordance with the PRISMA 2020 guidelines [5] and reported following the Synthesis Without Meta-analysis (SWiM) recommendations [6], and was prospectively registered in PROSPERO (CRD420261415084). During title-level screening, a generative artificial intelligence tool (large language model) supported the exclusion of records clearly outside the review scope; all AI-flagged records were manually verified by two reviewers before final exclusion. The tool was not used for data extraction, quality assessment, or manuscript writing.
