# Supplementary material for: Timing of aneurysm repair and clinical outcomes after aneurysmal subarachnoid hemorrhage: a systematic review
Source: J Neurol. 2026 Sep 9;273(10):582. doi: 10.1007/s00415-026-14063-x (PMC13553604; doi:10.1007/s00415-026-14063-x)
Supplement: Supplementary file 1 [file 415_2026_14063_MOESM1_ESM.docx]

**Supplementary Table S1**. GRADE certainty of evidence for the association between onset-to-treatment time and mortality in aneurysmal subarachnoid hemorrhage

| **Domain** | **Rating** | **Reason** |
| --- | --- | --- |
| Study design | Observational studies; starting certainty: low | Observational evidence started at low certainty. Serious risk of bias reduced the rating to very low, the lowest GRADE category; serious inconsistency and imprecision further reinforced this judgment. No upgrading factors were identified. |
| Risk of bias | Serious | Confounding by indication, survivor and treated-survivor bias, and selective inclusion in timing analyses were frequent and could not be fully addressed by conventional multivariable or propensity-based adjustment. |
| Inconsistency | Serious | Findings were conflicting: two timing-based analyses favored earlier treatment, three favored later treatment, four found no difference, and three reported non-significant numerical trends. |
| Indirectness | Not serious | The included populations, exposure, comparisons, and mortality outcomes directly addressed the review question. |
| Imprecision | Serious | Several estimates had wide confidence intervals, and heterogeneity of timing categories and effect measures precluded calculation of a pooled overall estimate. |
| Publication bias | No additional downgrade | The small number of studies and absence of a pooled estimate precluded formal assessment; no additional downgrade was applied. |
| Upgrading factors | None identified | No sufficiently robust large effect, dose-response gradient, or residual-confounding pattern justified rating up the certainty. |
| Overall certainty | Very low (⊕◯◯◯) | Observational evidence started at low certainty and was downgraded to very low because of serious risk of bias. Serious inconsistency and imprecision further reinforced the very-low-certainty judgment but could not reduce it below the lowest GRADE category. |

**Supplementary Table S2.** Complete database-specific search strategies.

| **Database** | **Date last searched** | **Complete search strategy** |
| --- | --- | --- |
| **PubMed /MEDLINE** | 18 April 2026 | ("subarachnoid aneurysm"[TITLE/ABSTRACT] OR "Subarachnoid Hemorrhage"[MeSH] OR "subarachnoid hemorrhage"[TITLE/ABSTRACT] OR "aneurysmal SAH"[TITLE/ABSTRACT] OR "aneurysmatic SAH"[TITLE/ABSTRACT] OR "ruptured intracranial aneurysms"[TITLE/ABSTRACT] OR "Aneurysmatic subarachnoid hemorrhage"[TITLE/ABSTRACT] OR "aSAH"[TITLE] OR "aneurysmal subarachnoid haemorrhage"[TITLE/ABSTRACT] OR "HSAa"[TITLE/ABSTRACT]) AND ("coiling"[tw] OR "endovascular"[tw] OR "coil"[tw] OR "Surgery"[tw] OR "Microsurgery"[tw] OR "clip"[tw] OR "clipping"[tw] OR "timing"[tw] OR "early"[tw] OR "subacute"[tw] OR "acute"[tw] OR "time to treatment"[tw] OR "treatment delay"[tw] OR "early intervention"[tw] OR "delay"[tw] OR "onset to treatment"[tw] OR "onset"[tw] OR "onset-treatment"[tw] OR "door to treatment"[tw] OR "time to aneurysm"[tw]) AND ("complications"[tw] OR "outcomes"[tw] OR "outcome"[tw] OR "mortality"[tw] OR "functional outcome"[tw] OR "vasospasm"[tw] OR "rebleeding"[tw] OR hydrocephalus[tw] OR impact[tw] OR "length of stay"[tw] OR "Glasgow Outcome Scale"[tw] OR "modified Rankin Scale"[tw]) NOT ("Trauma"[TITLE] OR "unruptured"[TITLE]) |
| **Scopus** | 17 April 2026 | ( TITLE-ABS-KEY ( "aneurysmal subarachnoid hemorrhage" OR "subarachnoid hemorrhage" OR "subarachnoid aneurysm" OR "aSAH" OR "ruptured intracranial aneurysm" OR "ruptured intracranial aneurysms" OR "aneurysmal SAH" OR "aneurysmal subarachnoid haemorrhage" ) AND TITLE-ABS ( "coiling" OR "coil" OR "clipping" OR "time to treatment" OR "treatment delay" OR "early intervention" OR "timing of treatment" OR "ictus to treatment" OR "onset to treatment" OR "timing" OR "treatment delayed" OR "delayed treatment" OR "onset-treatment" OR "door to treatment" OR "time to aneurysm" ) AND TITLE-ABS ( "functional outcome" OR "vasospasm" OR "rebleeding" OR "outcome" OR "outcomes" OR "length of stay" OR "modified Rankin Scale" ) AND NOT TITLE-ABS ( "review" OR "meta-analysis" OR "systematic" OR "unruptured" OR "trauma" OR "traumatic" OR "thrombectomy" OR "occlusive" OR "clazosentan" OR "nicardipine" OR "nimodipine" OR "dexamethasone" OR "predictors" OR "fasudil" OR "case-report" OR "case report" ) ) AND ( LIMIT-TO ( LANGUAGE , "English" ) ) AND ( LIMIT-TO ( DOCTYPE , "ar" ) ) AND ( LIMIT-TO ( EXACTKEYWORD , "Subarachnoid Hemorrhage" ) ) AND PUBYEAR > 2014 AND PUBYEAR < 2027 |
| **LILACS** | 15 April 2026 | (tw:(hemorragia subaracnóidea OR hemorragia subaracnoidea OR subarachnoid hemorrhage OR aneurysmal SAH OR ruptured intracranial aneurysm)) AND (tw:(tempo para tratamento OR atraso no tratamento OR intervenção precoce OR time to treatment OR treatment delay OR early intervention OR tiempo al tratamiento OR demora en el tratamiento OR intervención temprana OR ictus)) AND (tw:(mortalidade OR mortalidad OR mortality OR desfecho funcional OR resultado funcional OR functional outcome OR vasoespasmo OR vasospasm OR ressangramento OR rebleeding OR hidrocefalia OR hydrocephalus OR tempo de internação OR length of stay)) |

**Note.** The following limits were applied to all databases: publication date from 1 January 2015 to the database-specific last-search date; English, Portuguese, or Spanish language; exclusion of review articles and nonhuman animal studies.

**Supplementary Table S3.** Exclusion criteria for the use of AI for title-screening.

| **Domain** | **Exclusion criteria** | **Rationale / examples** |
| --- | --- | --- |
| Population | Not aneurysmal SAH | Traumatic SAH, unruptured intracranial aneurysms, hemorrhage from arteriovenous malformations, vasculitis, or other causes; non-human studies |
| Population | Pediatric population | Patients aged < 18 years |
| Intervention | No defined onset-to-treatment interval | Studies comparing treatment modalities (clipping vs. coiling) without timing analysis; studies on perioperative management only |
| Intervention | No aneurysm repair | Conservative or palliative management only; autopsy studies |
| Comparator | Absence of temporal comparison | Single-arm studies without stratification or analysis by time interval |
| Outcome | No clinical outcomes of interest | Studies reporting technical/procedural measures, biomarkers, imaging findings, or quality of life without functional scale or mortality data |
| Study type | Non-original research | Narrative reviews, systematic reviews or meta-analyses without original patient data, editorials, letters, commentaries, guidelines |
| Study design | Duplicate publication | Same cohort reported in multiple articles without additional outcome data |

**Note:** The criteria below were translated into a structured PICO-based prompt submitted to the generative AI model (Anara AI). For each title and its available metadata, the model evaluated whether any exclusion criterion was met and returned a binary flag (“exclude” / "retain") with the rationale. All AI-flagged records were then manually verified by two reviewers before final exclusion.

**Supplementary Table S4. Domain-based methodological assessment of the included observational timing studies**

| Study | Domain | Judgment | Timing-specific concerns |
| --- | --- | --- | --- |
| Brawanski et al. (2020) | Cohort representativeness | Low concern | Timing analysis was restricted to 211/449 patients with reliably documented ictus time who underwent aneurysm treatment. No adjusted timing estimate or baseline comparison across timing groups was reported. Major concerns include confounding by indication, survivor/treated-survivor bias, referral and treatment-era effects, and inconsistent reporting of the third timing interval. |
|  | Selection into timing analysis | High concern |  |
|  | Timing ascertainment | Some concerns |  |
|  | Adjustment for clinical severity | High concern |  |
|  | Additional confounder adjustment | High concern |  |
|  | Outcome assessment | Some concerns |  |
|  | Follow-up/attrition | Low concern |  |
| Buscot et al. (2022) | Cohort representativeness | Low concern | Consecutive multicenter cohort with comprehensive adjustment and sensitivity analyses, but restricted to treated patients; 93/575 untreated patients were excluded and 86% died. The discharge analysis additionally conditioned on hospital survival. Residual confounding by indication and survivor/treated-survivor selection remain possible; symptom-onset proxies were used in 47 patients, and the adjusted mortality nadir was data-derived despite a nonsignificant nonlinear association. |
|  | Selection into timing analysis | High concern |  |
|  | Timing ascertainment | Some concerns |  |
|  | Adjustment for clinical severity | Some concerns |  |
|  | Additional confounder adjustment | Some concerns |  |
|  | Outcome assessment | Some concerns |  |
|  | Follow-up/attrition | Low concern |  |
| Dellaretti et al. (2018) | Cohort representativeness | Some concerns | Retrospective clipping-only cohort restricted to patients surviving until referral and surgery; treatment timing primarily reflected delayed admission. No comprehensive confounder adjustment was performed. Timing-group sizes and the reference category were inconsistently reported, and functional outcome was assessed only at hospital discharge. |
|  | Selection into timing analysis | High concern |  |
|  | Timing ascertainment | High concern |  |
|  | Adjustment for clinical severity | High concern |  |
|  | Additional confounder adjustment | High concern |  |
|  | Outcome assessment | Some concerns |  |
|  | Follow-up/attrition | Low concern |  |
| Hostettler et al. (2023) | Cohort representativeness | Low concern | Prospective hospital registry analyzed retrospectively, but restricted to treated patients and explicitly excluding patients who died before aneurysm occlusion. Marked baseline severity imbalance indicates substantial confounding by indication. Multivariable models adjusted for age and major hemorrhage-severity measures, but did not consistently account for transfer, treatment modality, diagnostic delay, or other determinants of timing. Exact timing in hours was available for only 535/853 patients, and functional outcome was assessed at discharge. |
|  | Selection into timing analysis | High concern |  |
|  | Timing ascertainment | Some concerns |  |
|  | Adjustment for clinical severity | High concern |  |
|  | Additional confounder adjustment | Some concerns |  |
|  | Outcome assessment | Some concerns |  |
|  | Follow-up/attrition | Low concern |  |
| Koester et al. (2023) | Cohort representativeness | Some concerns | The analysis was restricted to definitively treated patients and excluded patients who reruptured or died before transfer, as well as untreated patients, creating substantial survivor and treated-survivor selection. Rupture time was estimated from patient or family reports, and treatment timing was determined without a standardized protocol and could be influenced by physician decisions, weekend admission, bypass requirements, intracranial pressure, and clinical urgency. Propensity adjustment included major measured covariates but did not fully address these determinants. Only 391/1013 patients had at least 180 days of follow-up, with particularly limited follow-up among ultra-early endovascular patients. |
|  | Selection into timing analysis | High concern |  |
|  | Timing ascertainment | Some concerns |  |
|  | Adjustment for clinical severity | Some concerns |  |
|  | Additional confounder adjustment | Some concerns |  |
|  | Outcome assessment | Some concerns |  |
|  | Follow-up/attrition | High concern |  |
| Kurisu et al. (2023) | Cohort representativeness | Some concerns | The analysis was restricted to treated patients, and patients treated after 24 h necessarily had to survive and remain eligible until aneurysm repair. Most delayed cases resulted from delayed presentation, selecting patients with milder baseline severity and creating substantial survivor/treated-survivor bias. Marked WFNS imbalance remained despite adjustment, and reasons for delay, transfer, missed diagnosis, physician-driven treatment selection, and pre-existing vasospasm were not fully controlled. The adjusted outcome table also contained inconsistent outcome labeling. |
|  | Selection into timing analysis | High concern |  |
|  | Timing ascertainment | Some concerns |  |
|  | Adjustment for clinical severity | Some concerns |  |
|  | Additional confounder adjustment | High concern |  |
|  | Outcome assessment | Some concerns |  |
|  | Follow-up/attrition | Low concern |  |
| Lage et al. (2020) | Cohort representativeness | Low concern | The analysis was restricted to patients who survived and underwent microsurgical clipping within 72 h, excluding untreated, endovascularly treated, and later-treated patients. Adjustment included only dichotomized WFNS grade and did not account for detailed clinical severity, transfer and diagnostic delays, hospital logistics, aneurysm characteristics, or treatment era. Because the ultra-early protocol was implemented progressively over a 17-year period, secular changes in care may have confounded the observed association. |
|  | Selection into timing analysis | High concern |  |
|  | Timing ascertainment | Some concerns |  |
|  | Adjustment for clinical severity | High concern |  |
|  | Additional confounder adjustment | High concern |  |
|  | Outcome assessment | Some concerns |  |
|  | Follow-up/attrition | Low concern |  |
| Loconi-Vallejos et al. (2023) | Cohort representativeness | Some concerns | The analysis was restricted to surgically clipped patients who survived and remained eligible until treatment, with only 11 patients in the 1-3-day reference group. Timing was retrospectively defined from recognition of the vascular event rather than through a clearly standardized ictus-time ascertainment procedure. The adjusted model included numerous covariates despite only 30 poor-outcome events and incorporated potentially post-exposure variables such as rebleeding, delayed cerebral ischemia, and nosocomial infection, raising concerns about overfitting and overadjustment. Fourteen patients with missing exposure or outcome data were excluded. |
|  | Selection into timing analysis | High concern |  |
|  | Timing ascertainment | Some concerns |  |
|  | Adjustment for clinical severity | Some concerns |  |
|  | Additional confounder adjustment | High concern |  |
|  | Outcome assessment | Some concerns |  |
|  | Follow-up/attrition | Some concerns |  |
| Lu et al. (2023) | Cohort representativeness | Some concerns | The analysis was restricted to treated poor-grade patients, and patients in the later timing groups necessarily had to survive and remain eligible until aneurysm repair. The report did not characterize untreated patients or pretreatment deaths and reruptures in the source poor-grade population. Although models adjusted for measured clinical severity and aneurysm characteristics, reasons for delay, transfer, clinical instability, pre-existing vasospasm, treatment eligibility, and other determinants of timing were not controlled. Only seven patients were treated after day 10, resulting in highly unstable estimates. |
|  | Selection into timing analysis | High concern |  |
|  | Timing ascertainment | Some concerns |  |
|  | Adjustment for clinical severity | Some concerns |  |
|  | Additional confounder adjustment | High concern |  |
|  | Outcome assessment | Some concerns |  |
|  | Follow-up/attrition | Low concern |  |
| Ohbuchi et al. (2023) | Cohort representativeness | Low concern | Although the source cohort included consecutive high-grade patients and untreated cases, the primary timing analysis was restricted to patients who completed the composite “appropriate treatment” within 72 h. This exposure combined aneurysm occlusion with the final required ICP-control procedure, and the order of procedures was not considered. The 13-h threshold was selected using the outcome data and tested in the same cohort without external validation. Subjective treatment selection, reasons for delay, center effects, clinical instability, and other determinants of timing were not fully controlled. Twenty-seven patients lacked 3-month outcome data. |
|  | Selection into timing analysis | High concern |  |
|  | Timing ascertainment | Some concerns |  |
|  | Adjustment for clinical severity | Some concerns |  |
|  | Additional confounder adjustment | High concern |  |
|  | Outcome assessment | Some concerns |  |
|  | Follow-up/attrition | Some concerns |  |
| Şimsek and Akinci (2022) | Cohort representativeness | Some concerns | The main analyses compared 34 patients undergoing clipping within 6 h with 178 patients who either underwent later clipping or received no clipping, thereby conflating treatment timing with receipt of treatment. The cohort definition was internally inconsistent because the eligibility criteria required surgical clipping, whereas 116 patients without clipping were included. Timing groups differed substantially in age, WFNS grade, and Fisher grade, but the regression included only ultra-early surgery and did not adjust for these factors. A major protocol change and continuous CTA availability after 2011 created substantial treatment-era confounding. |
|  | Selection into timing analysis | High concern |  |
|  | Timing ascertainment | Some concerns |  |
|  | Adjustment for clinical severity | High concern |  |
|  | Additional confounder adjustment | High concern |  |
|  | Outcome assessment | Some concerns |  |
|  | Follow-up/attrition | Low concern |  |
| Teo et al. (2017) | Cohort representativeness | Some concerns | Although derived from a multicenter randomized trial, treatment timing was not randomized. The post hoc timing analysis was restricted to patients receiving a single aneurysm-treatment modality and excluded untreated patients, while the parent trial had already excluded patients without a reasonable prospect of survival. The crude timing association was reported to disappear after adjustment, but no adjusted timing effect estimate was presented. Reasons for treatment delay and center-level or logistical determinants were not controlled, and delayed cerebral ischemia was included despite being a potential post-treatment mediator. The timing-outcome analysis included 749 of 767 single-modality patients without explaining the remaining 18. |
|  | Selection into timing analysis | High concern |  |
|  | Timing ascertainment | Some concerns |  |
|  | Adjustment for clinical severity | Some concerns |  |
|  | Additional confounder adjustment | High concern |  |
|  | Outcome assessment | Low concern |  |
|  | Follow-up/attrition | Some concerns |  |
| Tykocki et al. (2017) | Cohort representativeness | Some concerns | The analysis was restricted to treated poor-grade patients, and those treated after 24 h necessarily had to survive and remain eligible until aneurysm repair. Early and late groups differed markedly in age, intracerebral hematoma, and midline shift, and the authors acknowledged physician-driven selection for earlier treatment. Although multivariable adjustment was reported, the model presentation contains internally incompatible coefficients, odds ratios, confidence intervals, and p values and includes complementary exposure and modality categories without a clearly defined reference. Functional outcome was assessed only at hospital discharge. |
|  | Selection into timing analysis | High concern |  |
|  | Timing ascertainment | Some concerns |  |
|  | Adjustment for clinical severity | High concern |  |
|  | Additional confounder adjustment | High concern |  |
|  | Outcome assessment | Some concerns |  |
|  | Follow-up/attrition | Low concern |  |
| Vergouwen et al. (2023) | Cohort representativeness | Low concern | Prospectively collected multicenter data provided detailed timing and standardized 6-month outcome assessment, but the analysis was restricted to patients treated within 24 h and excluded untreated and later-treated patients. The <6-h group had substantially worse clinical and radiological severity and more rebleeding. Treatment timing was also strongly related to daytime versus nighttime presentation. Although predefined adjustment and additional rebleeding analyses were performed, confounding by indication, logistical confounding, and survivor/treated-survivor selection remain important concerns. |
|  | Selection into timing analysis | High concern |  |
|  | Timing ascertainment | Low concern |  |
|  | Adjustment for clinical severity | Some concerns |  |
|  | Additional confounder adjustment | High concern |  |
|  | Outcome assessment | Low concern |  |
|  | Follow-up/attrition | Low concern |  |
| Wang B. et al. (2025) | Cohort representativeness | Low concern | The multicenter source cohort was consecutively assembled, but the timing analysis excluded 380 untreated patients, including 283/335 patients with available follow-up who died before aneurysm repair, and 205/1209 treated patients were lost to follow-up. Later-treatment groups were therefore conditioned on survival and continued treatment eligibility. The <12-h group had substantially more WFNS grade V and Fisher grade IV disease. Although adjustment included WFNS, Fisher grade, treatment modality, and selected comorbidities, reasons for delay, clinical instability, transfer and center logistics, rebleeding, and pretreatment supportive procedures were not adequately controlled. Symptom-onset time was retrospectively reconstructed to the hour, and functional outcomes were primarily assessed by telephone. |
|  | Selection into timing analysis | High concern |  |
|  | Timing ascertainment | Some concerns |  |
|  | Adjustment for clinical severity | Some concerns |  |
|  | Additional confounder adjustment | High concern |  |
|  | Outcome assessment | Some concerns |  |
|  | Follow-up/attrition | High concern |  |
| Wang B. et al. (2026) | Cohort representativeness | Low concern | The analysis was restricted to patients who underwent aneurysm treatment within 72 h and excluded untreated patients, later-treated patients, those with severe comorbidities, and patients receiving both treatment modalities. Later timing groups were therefore conditioned on survival and continued treatment eligibility. Marked gradients in WFNS grade, Hunt–Hess grade, and ICH/IVH indicate substantial confounding by indication. Although the models adjusted for several severity measures, reasons for delay, transfer, center and logistical factors, clinical instability, and pre-treatment events were not adequately controlled. The 32.6-h mortality nadir was data-derived without external validation, symptom onset was patient- or proxy-reported, and 17.9% of patients were lost to follow-up. |
|  | Selection into timing analysis | High concern |  |
|  | Timing ascertainment | Some concerns |  |
|  | Adjustment for clinical severity | Some concerns |  |
|  | Additional confounder adjustment | High concern |  |
|  | Outcome assessment | Some concerns |  |
|  | Follow-up/attrition | High concern |  |
| Wu et al. (2022) | Cohort representativeness | Some concerns | The single-center analysis was restricted to 86 endo-vascularly treated high-grade patients and excluded patients undergoing clipping or conservative management. Patients treated after 24 h necessarily had to survive and remain eligible for aneurysm repair. Hunt–Hess grade differed substantially between timing groups, while radiological severity, pre-treatment neurological deterioration, re-bleeding, reasons for delay, transfer, and logistical determinants were not controlled. The adjusted model included postoperative hydrocephalus and pulmonary infection, which may be post-exposure variables, and the 12.5-h cutoff was selected from the same small cohort without validation. |
|  | Selection into timing analysis | High concern |  |
|  | Timing ascertainment | Some concerns |  |
|  | Adjustment for clinical severity | Some concerns |  |
|  | Additional confounder adjustment | High concern |  |
|  | Outcome assessment | Low concern |  |
|  | Follow-up/attrition | Low concern |  |
| Zhang F. et al. (2017) | Cohort representativeness | Some concerns | The analysis was restricted to patients who underwent successful endovascular coiling and excluded clipping, untreated cases, and patients with incomplete records; the report further states that patients with poor outcomes attributed to the coiling procedure were excluded. Later-treated patients therefore had to survive and remain eligible until aneurysm repair. Timing was retrospectively classified using overlapping “within 12 h” and “≥12 h” categories, without a described ictus-time ascertainment procedure or baseline comparison across timing groups. Although Hunt–Hess and Fisher grades were included, reasons for delay and other clinical or logistical confounders were not controlled. Regression coefficients were inconsistent with the reported odds ratios, and 3-month GOS ascertainment and missing-data exclusions were insufficiently described. |
|  | Selection into timing analysis | High concern |  |
|  | Timing ascertainment | Some concerns |  |
|  | Adjustment for clinical severity | Some concerns |  |
|  | Additional confounder adjustment | High concern |  |
|  | Outcome assessment | Some concerns |  |
|  | Follow-up/attrition | Some concerns |  |
| Zhang X. et al. (2021) | Cohort representativeness | Some concerns | The single-center cohort was restricted to patients who reached a high-volume referral hospital and underwent endovascular treatment, thereby excluding untreated patients, patients managed with clipping, and patients who died before aneurysm repair. Later treatment intervals were consequently conditioned on survival, referral, and continued eligibility for treatment. Timing was retrieved retrospectively and could only be classified in days because precise times were unavailable for all patients. Although the authors qualitatively reported that shorter onset-to-treatment time was associated with poorer discharge outcome, no timing-specific outcome counts, effect estimate, or p value was provided; the multivariable outcome model instead evaluated onset-to-admission time. Strong severity-related referral patterns and unmeasured transfer, diagnostic, and logistical factors limit causal interpretation. |
|  | Selection into timing analysis | High concern |  |
|  | Timing ascertainment | Some concerns |  |
|  | Adjustment for clinical severity | High concern |  |
|  | Additional confounder adjustment | High concern |  |
|  | Outcome assessment | Some concerns |  |
|  | Follow-up/attrition | Low concern |  |
| Zhao et al. (2015) | Cohort representativeness | Some concerns | The analysis was restricted to persistently poor-grade patients selected for microsurgical clipping; patients who died after resuscitation, had care withdrawn, remained untreated, or underwent endovascular treatment were excluded. Patients in the delayed group had to survive and remain eligible for surgery for up to 21 days. The <24-h group had substantially more WFNS grade V disease, brain herniation, and intracerebral hematoma. Adjustment for these severity measures attenuated the crude association, but age, aneurysm location, referral and logistical delays, center effects, family decision-making, and other determinants of timing were not included. Ictus was defined as loss of consciousness, and functional outcome was assessed at variable follow-up times ranging from 6 to 28 months. |
|  | Selection into timing analysis | High concern |  |
|  | Timing ascertainment | Some concerns |  |
|  | Adjustment for clinical severity | Low concern |  |
|  | Additional confounder adjustment | High concern |  |
|  | Outcome assessment | Some concerns |  |
|  | Follow-up/attrition | Low concern |  |

**Note.** Judgments refer specifically to the validity of the association between onset-to-treatment time and clinical outcomes, rather than to the overall quality of each publication. No total scores or global low-, moderate-, or high-risk classifications were assigned. “Some concerns” indicates limitations that may affect the estimate but were not sufficient to support a high-concern judgment.
